# High-Performance Boron Nitride-Based Membranes for Water Purification

**DOI:** 10.3390/nano12030473

**Published:** 2022-01-29

**Authors:** Natalia García Doménech, Finn Purcell-Milton, Adrián Sanz Arjona, Maria-Luisa Casasín García, Maeve Ward, Marc Brunet Cabré, Aran Rafferty, Kim McKelvey, Peter Dunne, Yurii K. Gun’ko

**Affiliations:** 1 School of Chemistry, Trinity College Dublin, D02 PN40 Dublin, Ireland; garciadn@tcd.ie (N.G.D.); PURCELFI@tcd.ie (F.P.-M.); SANZARJA@tcd.ie (A.S.A.); maria.luisa.casasin@gmail.com (M.-L.C.G.); mward5@tcd.ie (M.W.); brunetcm@tcd.ie (M.B.C.); rafferta@tcd.ie (A.R.); p.w.dunne@tcd.ie (P.D.); 2 BiOrbic, Bioeconomy Research Centre, University College Dublin, D04 V1W8 Dublin, Ireland; 3 School of Chemical and Physical Sciences, Victoria University of Wellington, Wellington 6012, New Zealand; kim.mckelvey@vuw.ac.nz

**Keywords:** nanofiltration, membranes, water purification, sustainable, separation technologies, 2D nanomaterials

## Abstract

In recent years, nanotechnology-based approaches have resulted in the development of new alternative sustainable technologies for water purification. Two-dimensional (2D) nanomaterials are an emerging class of materials for nanofiltration membranes. In this work, we report the production, characterisation and testing of a promising nanofiltration membrane made from water-exfoliated boron nitride (BN) 2D nanosheets. The membranes have been tested for water purification and removal of typical water-soluble dyes such as methyl orange, methylene blue and Evans blue, with the water-exfoliated BN membranes achieving retention values close to 100%. In addition, we compared the performance of membranes made from water-exfoliated BN with those produced from BN using sonication-assisted liquid exfoliation in selected organic solvents such as 2-propanol and N-methyl-2-pyrrolidone. It was found that membranes from the water-exfoliated BN showed superior performance. We believe this research opens up a unique opportunity for the development of new high-performance environmentally friendly membranes for nanofiltration and new sustainable separation technologies.

## 1. Introduction

Separation methods using membranes play a key role in industrial processes such as water treatment [1] and purification of active pharmaceutical and food ingredients [2] due to their high efficiency, low energy use, convenience for up- or down-scaling and possibility of continuous flow operation. Recently, nanotechnology-based approaches, such as nanofiltration (NF), have emerged as possibly superior and cost-effective means to eliminate sediments, chemical species, pathogens, toxins and impurities [3,4,5]. NF is a type of filtration that uses membranes with pore size between 0.5 and 10 nm and a working pressure between 5 and 40 bar, and is frequently used for the filtration of solid dust, liquid droplets, sugars, proteins, dyes, multivalent ions and microorganisms (such as viruses and bacteria). NF is often considered as an intermediate between reverse osmosis and ultrafiltration, rejecting molecules with sizes in the order of a nanometre [6,7]. NF presents intrinsic properties such as high permeation to monovalent ions, low permeation to divalent ions and higher flux than reverse osmosis membranes. Due to these features, NF has been adopted for several important applications, such as water treatment and purification technologies used in biopharmaceutical and food industries [8].

Nanomaterials are potentially excellent building blocks for NF membranes. The most commonly used nanomaterials are nanoparticles (often metal and metal oxide) and nanotubes, usually carbon nanotubes. Nanoparticles tend to be mixed with various polymeric matrixes to improve membrane properties, such as flux and rejection [9,10,11,12]. Carbon nanotubes (CNTs) are frequently added to mixed polymeric matrixes to improve separation and antifouling properties [13,14,15,16]. In recent years, two-dimensional (2D) nanomaterials have gained interest as building blocks for NF membranes. These materials offer the option of high chemical inertness, as found in ceramics, combined with the ease of processability of polymers. Unique to 2D materials such as graphene and boron nitride is a very high breaking strength, and single layers have been shown to tolerate more than 20% elastic distortion, presenting exceptionally large flexibility [17]. In addition, these materials have a very large total surface area due to their intrinsic layered nature [18,19].

Boron nitride (BN) is a 2D material that has received a lot of attention in recent years, and is often regarded as a white counterpart of graphite, with the boron and nitrogen atoms appearing where the carbon atoms do in its analogue [20]. BN is a very attractive 2D nanomaterial for NF, as it is inexpensive, environmentally friendly, chemically stable, mechanically strong and electrically insulating due to its band gap of around 5–6 eV [20,21,22,23]. It has also been reported to show a distinctly unique property of having high thermal conductivity while being an electrical insulator, an emergent property due to the nanostructure of the material, which offers a unique design capability [20,24,25,26].

BN-based membranes have previously been reported for potential use in organic molecule separation and pharmaceutical applications [26,27,28,29,30,31,32,33]. BN nanosheets have several advantages over established materials for separation and purification, due to their high surface area, nanosheet structure and polarity of their bonds [30,34], making BN nanostructures sufficient adsorbents of various substances, ranging from organic pollutants to hydrogen molecules [28,35,36]. Recent examples reported in the literature include the use of BN materials for water treatment to remove [30,37,38] organic (ranging from oils, solvents and dyes) [28,34,35,39,40,41,42] and inorganic pollutants (such as heavy metals) [41,43,44,45,46,47,48] as well as pharmaceutical components from water [29,49]. BN-based membranes can be broadly categorised into membranes formed from a mixture of polymer and BN, and membranes made from pure BN. Naturally, the major advantage of pure BN filters is their stability across a large temperature, chemical and pH range, whereas membranes that incorporate polymers benefit from ease of use and more wide-ranging mechanical properties.

There are several ways to produce exfoliated 2D BN materials, with most based on a top-down approach, including micromechanical cleavage, chemical exfoliation, mechanical exfoliation (most commonly ball milling) and sonication-assisted liquid-phase exfoliation [50]. Each approach has advantages and disadvantages, with the most successful to date in the field of membrane preparation being based on either ball milling or liquid-phase exfoliation. In the case of liquid exfoliation, it has been shown that exfoliation in bulk can be performed in common solvents, yielding mono- and low-number layers. The method is insensitive to ambient conditions and shows potential for scale-up [51]. In addition, BN presents an extra advantage, as it can be dispersed in water due to sonication-assisted hydrolysis [52]. Solvent exfoliation is one of the preferred methods for 2D nanosheet production as it is a simple procedure and does not use a third-phase dispersant, such as a surfactant [53]. Some solvents are capable of providing sufficient external energy to reduce the interlayer binding, which keeps the nanosheets together, and expanding the stacking distance between the different layers [53]. Afterwards, depending on the ability of the solvent to stabilise the layers, the bulk material can be exfoliated. Nevertheless, the reverse process (re-aggregation of the nanosheets) could occur due to their interlayer binding energy. Therefore, when it comes to liquid-phase exfoliation, it is key to choose the correct solvent that will promote the exfoliation and suspension of the nanosheets in the solvent [54]. Selection of solvents, however, is frequently carried out based on trial-and-error methods and experience [54]. Coleman et al. have proposed that in order to obtain a stable dispersion of the exfoliated material, the solvent of choice has to match the surface energy of the nanosheet and, thus, minimise the enthalpy of mixing [55].

Due to the polar nature of BN, the preferential choice has been a polar solvent for sonication-assisted exfoliation such as dimethylformamide (DMF) [56,57], dimethyl sulfoxide (DMSO) [57], N-methyl-2-pyrrolidone (NMP) [51,56,58], isopropanol (IPA) [51,59] and even water [60]. However, the best results for exfoliation of BN have so far been achieved in NMP [51]. In addition, the sonication-assisted exfoliation of BN is well suited for the production of BN-based membranes via the use of vacuum filtration, which is an effective method for membrane fabrication [26,32,49,61].

In this study, for the first time, we compare three commonly used solvents—NMP, IPA and water—for sonication-assisted liquid phase exfoliation of BN, and demonstrate the role they play in the resulting performance aspects of vacuum filtration-produced BN membranes. We demonstrate a low-cost, green and scalable process, using water as the solvent of choice to produce a novel BN-based membrane, and fully characterised it, demonstrating impressive retention across a number of dyes. We found that the level of exfoliation achieved during sonication is crucial to producing an effective membrane. We also specifically emphasize the use of water for exfoliation as an approach to achieve the most effective membranes.

## 2. Experimental Section

### 2.1. Materials

Boron nitride (h-BN) powder was purchased from Merck (particle size = 6–30 µm). N-methyl-2-pyrrolidone, (99%, HPLC) Evans blue (≥75%) and methylene blue (≥82%) were obtained from Sigma Aldrich (St. Louis, MO, USA). Methyl orange (≥95%) was purchased from VWR international Ltd. (Radnor, PA, USA). Isopropyl alcohol was obtained from Fischer (99.5%). Deionised water was obtained using a Milli-Q system with resin filters; this filtration was carried out in Trinity College laboratories. All solvents were analytically pure and used without further purification. Omnipore membrane filters (hydrophilic polytetrafluoroethylene, PTFE, with 20 nm pore size and 47 mm diameter) were purchased from Merck Millipore Limited (Burlington, MA, USA). The sonic bath used was Ultrawave model U100H from Ultrawave Ltd. (Cardiff, UK).

### 2.2. BN Exfoliation

First, 0.3 g of bulk BN (3 mg/mL) was dispersed in 100 mL of the chosen solvent: N-methyl-2-pyrrolidone (NMP), isopropyl alcohol (IPA) or Millipore water. These solutions were then continuously sonicated for 48 h for NMP and IPA and 24 h for Millipore water (using an Ultrawave model U100H). The solution was then immediately used for membrane formation.

### 2.3. Preparation of Membranes

BN membranes were produced by vacuum filtration of the exfoliated BN solution through a hydrophilic polytetrafluoroethylene (PTFE) membrane (20 nm pore size, 47 mm diameter). In brief, 50 mL of the exfoliated BN solution was passed through the PTFE template under vacuum filtration. Once all the solution had passed through, the pump was left running for a further 10–15 min to obtain a dried membrane. When using NMP, the membranes were washed three times with acetone (3 × 30 mL) to remove residual NMP.

### 2.4. Characterisation

Powder X-ray diffraction (XRD) was carried out on a zero-background holder using a Bruker D2 Phaser 2nd Gen. Measurements were performed for 2θ between 10 and 80, with no fluorescence correction and a 0.01 increment per second. Scanning electron microscopy (SEM) images were obtained using a Zeiss Ultra Plus Scanning Electron Microscope (Jena, Germany). High-resolution transmission electron microscopy (HRTEM) and scanning transmission electron microscopy (STEM) high-angle annular dark-field imaging (HAADF) were carried out using the FEI Titan operating at a beam voltage of 300 kV. UV-Vis spectra were recorded using a Cary 60 spectrophotometer with a wavelength between 200 and 800 nm. AFM measurements were carried out on a Park NX10 (Park Systems, Suwon, Korea). The AFM images were obtained in a non-contact mode (NCM) with a PPP-NCHR cantilever type (force constant of 42 N/m and resonance frequency of 330 kHz, Nanosensors). Single BN flakes were deposited by drop casting 0.03 mg/mL of exfoliated BN solution on gold-coated glass slides purchased from Evaporated Metal Films (TS-TA-134). BN-NMP samples were dried in a vacuum oven at 200 °C for 2 h prior to AFM measurement. BET surface area analysis was performed using a Nova 2400e Surface Area Analyser (Quantachrome, Hampshire, UK). Membranes were cut to size using a blade. Prior to analysis, samples were de-gassed for 6 h at 200 °C under vacuum. The BJH method was used to calculate the pore size diameter and pore volume from the desorption branch of the isotherms. The BJH values presented here include pores in the range of 1–30 nm. Mercury porosimetry was performed using an Autoscan-33 Porosimeter (Quantachrome, Hampshire, UK) with a default contact angle of 140°. Raman spectra were recorded using a Renishaw Raman Microscope with a 785 nm laser, equipped with three lenses and an automated xyz stage. The measurements were taken using the laser at 100% power with an exposure of 60 from 1600 to 1100 cm^−1^. The FTIR spectra were recorded using a Perkin Elmer Spectrum 100 with Perkin Elmer Universal ATR Sampling Accessory. It consists of 4 recording iterations collected, summed and averaged. The full spectra wavelength range was from 4000 cm^−1^ to 500 cm^−1^ in steps of 2 cm^−1^.

### 2.5. Retention Tests

Three water-soluble dyes were selected for testing the membranes’ retention: Evans blue, methyl orange and methylene blue. The concentrations were chosen based on the maximum of absorbance, which was between 1 and 1.5 a.u, as presented in Table S1. These concentrations match those used in the literature [62].

First, 20 mL of the dye solution was passed through the membrane and a UV-Vis spectrum of the permeate was recorded. In order to correct the retention, as a template was used, 20 mL of the initial Evans blue solution was filtered through the PTFE template. Once the permeate was obtained, further concentration of it was required to collect an adequate UV-Vis spectrum; for this, vacuum evaporation (rotavapor) was carried out until only solid matter was present. Later, 3 mL of Millipore water was added, and this solution was placed on the cuvette; this concentrated solution was measured with a UV-Vis spectrometer.

In order to calculate the retention, the following formula was used as found in the literature [63,64]:

$$R_{x}\left(\%\right)=\left(1-\frac{C_{P,X}}{C_{F,X}}\right)\cdot 100$$

where *R*_*x*_ is the retention in percentage, *C_F,X_* is the concentration of the analyte in the feed and *C_P,X_* is the concentration of the analyte in the permeate.

However, since concentration is proportionally correlated to the absorbance at a particular wavelength, the retention was calculated using the maximum absorbance. Therefore, the corrected formula was:

$$R_{x}\left(\%\right)=\left(1-\frac{A_{P,\lambda max}}{A_{F,\lambda max}}\right)\cdot 100$$

where *R_x_* is the retention in percentage, *A_F,λmax_* is the absorbance at 600 nm of the analyte in the feed and *A_P,λmax_* is the absorbance at 600 nm of the analyte in the permeate. The statistics of the retention were calculated using Origin software 2018.

## 3. Results and Discussion

### 3.1. Exfoliation of BN and Characterisation of BN Nanomaterials

**Figure 1:**
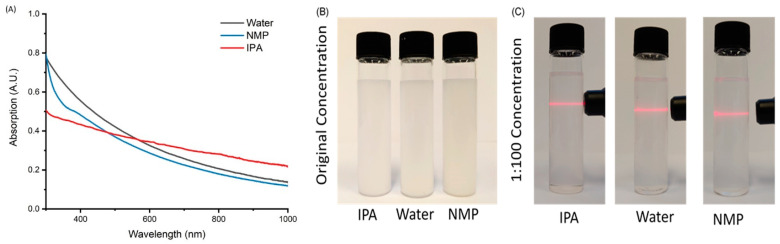
(**A**) UV-Vis spectra of exfoliated BN in NMP (red), IPA (blue) and water (green); (**B**) photograph of original solutions of BN exfoliated in the three solvents (3 mg/mL); (**C**) Tyndall effect on BN exfoliated in IPA, NMP and water (0.03 mg/mL).

In our work, the exfoliation of BN was carried out in three different solvents—water, IPA and NMP—to compare their performance with samples denoted as BN-Water, BN-IPA and BN-NMP, respectively. Samples were produced with a concentration of 3 mg/mL, which compares favourably with results reported in the literature using similar approaches [51,65,66,67], with the three solutions appearing very similar after exfoliation, with no distinct visible differences. UV-Vis spectra of the exfoliated BN solutions are presented in Figure 1, showing a decreased absorption at longer wavelengths and an increased absorption at shorter wavelengths. This can be related to the contribution of scatter and absorbance as the exfoliation extent of the samples increases. These results fit with reported spectra given in the literature [68]. All three samples clearly demonstrate the Tyndall effect, as shown in Figure 1, using diluted solutions of 0.03 mg/mL.

**Figure 2:**
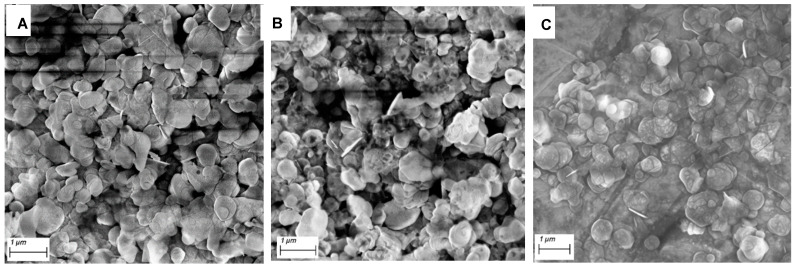
SEM images of exfoliated BN in (**A**) NMP, (**B**) IPA and (**C**) Millipore water where the nanosheets are present.

SEM images of the three BN samples are presented in Figure 2, comparing the nanosheets obtained (further images are given in the Supplementary Materials, see Figures S5–S7). The nanoflakes obtained are similar in appearance, with each sample exfoliated to a similar degree, especially when compared to SEM of non-exfoliated BN (see Supplementary Materials, Figure S4). The nanosheets obtained following exfoliation in NMP had a flake-size distribution of 0.548 ± 0.279 μm, while IPA had a mean size of 0.536 ± 0.178 μm and the water sample had a mean size of 0.581 ± 0.314 μm (Figures S1–S3). This indicates that the three solvents produce similar size nanosheet flakes with no significant differences between the samples under SEM.

**Figure 3:**
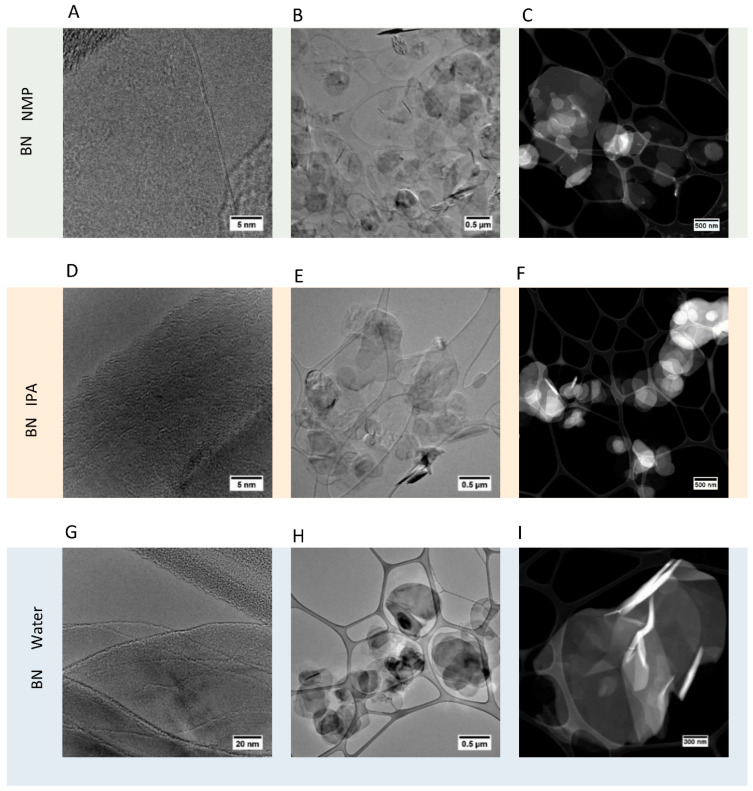
TEM (**A**,**B**,**D**,**E**,**G**,**H**) and STEM (**C**,**F**,**I**) images of 2D BN nanoflakes produced in NMP (**A**–**C**), IPA (**D**–**F**) and water (**G**–**I**).

Transmission electron microscopy (TEM) and scanning transmission electron microscopy (STEM) were carried out on the three samples of 2D BN, with the images shown in Figure 3 (additional images given in Supplementary Figures S8 and S9). This effectively confirms that exfoliation has taken place in each solvent case, with each sample showing minimal contrast, as well as strong transparency in TEM images, indicative of a thin exfoliated material. In addition, HRTEM analysis of the end of sheets confirmed that each flake comprised just a few monolayer sheets of BN. According to HRTEM imaging, the thickness of the thinnest individual BN layers after the exfoliation was in the range of 1–3 nm, corresponding to 3–9 monolayers of BN (one monolayer of BN is 0.33 nm).

STEM confirmed this information and allowed the clear round edge structure of the sheets to be clearly seen. A level of distinction could be made between the three different samples under STEM and TEM, with water samples showing the highest prevalence of single BN sheets or a low number of BN sheets. IPA showed the lowest prevalence of highly-exfoliated BN sheets, and NMP showed a degree of exfoliation between water and IPA.

**Figure 4:**
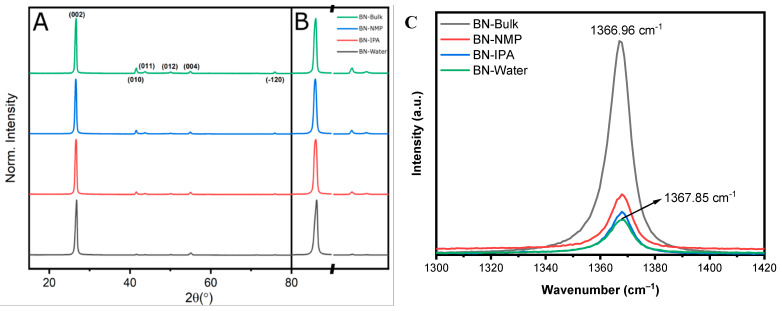
(**A**) Normalised PXRD patterns of bulk BN and exfoliated BN-NMP, BN-IPA and BN-Water. Miller indices are shown for the bulk material. (**B**) Comparison of (002), (010) and (011). (**C**) Raman spectra of h-BN in bulk form (black) and exfoliated BN-NMP (red), BN-IPA (blue) and BN-Water (green).

Powder XRD (PXRD) results are presented in Figure 4, showing the patterns obtained for the BN starting material (P −3 m 1, trigonal, a = 2.5100 Å, c = 6.6900 Å), designated as “bulk”, and the samples obtained from BN-NMP, BN-IPA and BN-Water. All four samples match perfectly with the BN model pattern, showing the (002), (010), (011), (012), (004) and (−120) peaks with decreasing intensity from left to right. In addition, no impurities can be observed. 

BN-NMP and BN-IPA samples show a lower crystallinity. Although peaks do become visually broader, they are not as prominent as for the water-exfoliated sample. It has been suggested that this improved exfoliation in water is due to the sonication-assisted hydrolysis of the solvent, helping the separation and dispersion of the BN nanosheets [60]. The dispersion between films is observed by comparing different peak intensities. Taking Miller indices (001) such as the most intense (002) and comparing it with (hk0) peaks, such as (010) or (011), it can be seen that after exfoliation, the relative intensity (I_(00l)_/I_(hk0)_) becomes larger. The related intensity of the (002) and (010) peaks gives a ratio (Ratio = I_(002)_/I_(010)_) of 1.26 for the bulk and 68.05 for the water-exfoliated sample, and this might be due to the random orientated stacking of films after drying. This has been previously reported by Bhimanapati and colleagues [69]. Due to exfoliation and later treatment, the layers stack on top of each other with a shifted angle along the c-axis. While there is a reduction in the intensity on the a- and b-axis, lowering the intensity due to random orientation, the c-axis keeps the same intensity. This effect of shifting along the vertical axis might generate the enhanced filtration of the material.

Raman spectra are shown in Figure 4C for the initial h-BN of the bulk material and the NMP, IPA and water-exfoliated samples. The peaks correspond to the Raman E_2g_ peak, with a bibliographic value of 1366 cm^−1^ [70]. This shows a characteristic decrease in intensity from the pristine material to the water-exfoliated one of 77% (Table S2). This is due to the reduction in layer thickness in the structure and is corroborated by the peak shift (Δν=0.89 cm^−1^), with the red shift of the peak reported to be due to the exfoliation of the multi-layered material. It is supported by the shortening of the B-N bond as a result of the absence of interlayer interactions due to exfoliation [67]. In addition, there is a substantial increase in the FWHM of 0.68 cm^−1^. This is attributed to a decrease in the overlapping of various peaks that form the characteristic peak due to a more heterogeneous orientation of the sheets that comprise the material. The Raman data presented here reach the same conclusion; that BN-Water shows a reduction in the homogeneous stacking of layers due to a more efficient exfoliation of BN, giving a reduction in thickness of the material.

FTIR spectra (Figure S11) show a broad band around 1370–1390 cm^−1^, corresponding to B-N stretching, and a slightly narrower band around 800–820 cm^−1^ as a result of B–N bending [71], characteristic of BN.

**Figure 5:**
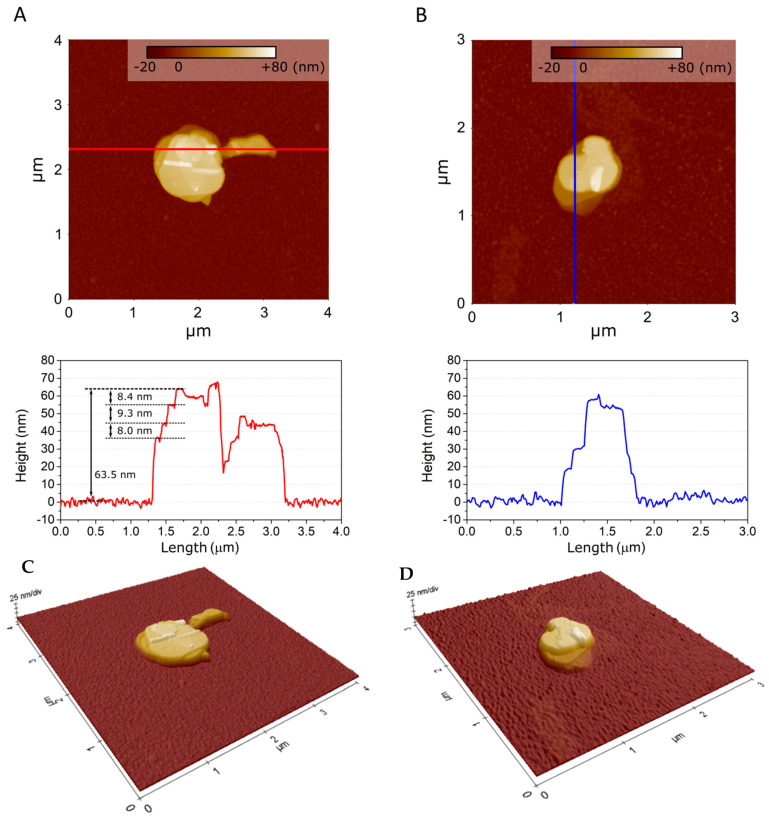
AFM images of (**A**) single BN flakes obtained from water exfoliation, with corresponding line profile; (**B**) BN flakes from NMP exfoliation, with corresponding line profile. Three-dimensional AFM images of single BN flakes obtained from (**C**) water exfoliation and (**D**) NMP exfoliation (images taken using 3× magnification on z-axis).

AFM images of single flakes of BN exfoliated in water, IPA and NMP are displayed in Figure 5 and Figure S10. The BN flake size ranges from hundreds of nm up to µm for water and NMP exfoliation. Only µm-size flakes were measured for exfoliation from IPA, as shown in Figure S10C. The flake size distribution corresponds to that measured from TEM data, presented in Figure S9. The morphology of the flakes is different in water, IPA and NMP. As observed in AFM images shown in Figure 5, water and NMP exfoliation produce flakes with characteristic steps and terraces. The line profiles, also displayed in Figure 5, show that step height between terraces is 8–9 nm, which corresponds to 24–27 monolayers of BN. This observation suggests that each BN flake exists within an oriented stack of BN layers. The flakes resulting from exfoliation with IPA media do not present such distinctive steps and terraces, as shown in Figure S10D. AFM images of single BN flakes reveal that while flake size is not significantly affected by a specific sonication media, the exfoliation with water or NMP could favour an oriented stacking of BN flakes.

### 3.2. Preparation and Characterisation of BN-Based Membranes

**Figure 6:**
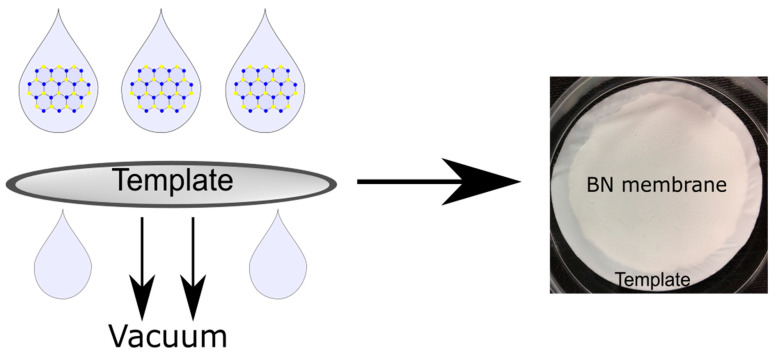
Schematic representation of BN membrane formation using vacuum filtration.

Following bulk BN exfoliation, the samples of 2D-BN, BN-Water, BN-NMP and BN-IPA were used to fabricate the respective membranes, denoted as BN-Water-Mem, BN-NMP-Mem and BN-IPA-Mem, respectively. The membranes were prepared using vacuum filtration, as described in the Methods section, and are shown in Figure 6. The resulting membranes were then characterised by light microscopy, SEM, AFM, BET surface area analysis and mercury porosimetry. A visual inspection revealed no significant differences between the membranes; images are provided in the Supplementary Materials.

**Figure 7:**
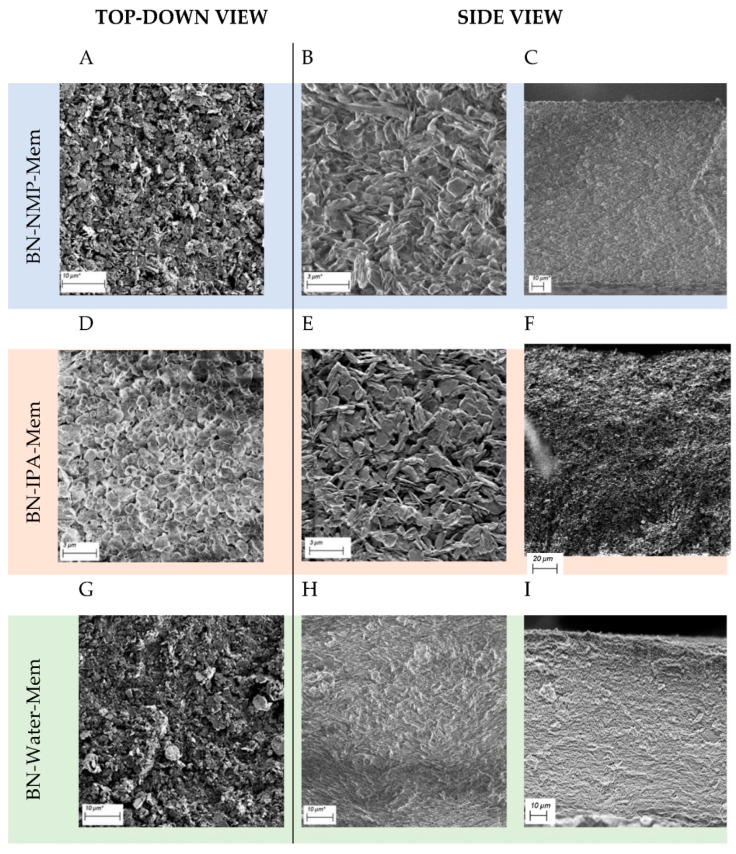
SEM of BN membranes with BN-NMP-Mem top-down view (**A**) and cross-sections (**B**,**C**); BN-IPA-Mem top-down view (**D**) and cross-sections (**E**,**F**); BN-Water-Mem top-down view (**G**) and cross-sections (**H**,**I**).

SEM images of each resulting membrane were taken and are presented in Figure 7. The top view of the three membranes (Figure 7A,D,G) showed little difference between the three solvents used. However, when viewing the cross-section of each, some distinct differences could be observed. Firstly, BN-IPA-Mem had a slightly increased mean thickness of 174.40 ± 1.60 µm (Table S4) compared to that found in BN-NMP-Mem and BN-Water-Mem, which showed thickness values of 148.90 ± 2.79 µm and 142.20 ± 3.55 µm, respectively.

Another observation was of differing packing when comparing the cross-sections of BN-NMP-Mem, BN-IPA-Mem and BN-Water-Mem (Figure 7C,F,I). BN-NMP-Mem and BN-IPA-Mem showed a more disorderly arrangement of the nanosheets (Figure 7C,F), whereas BN-Water-Mem (Figure 7I) displayed more stacked, ordered and strongly-aligned horizontal sheets of BN, with evidence of a wave-like perturbation in the stacking. This unique morphology is ascribed to the better exfoliation achieved using water. Additional cross-section and top-view images displayed in Figures S12–S14.

**Figure 8:**
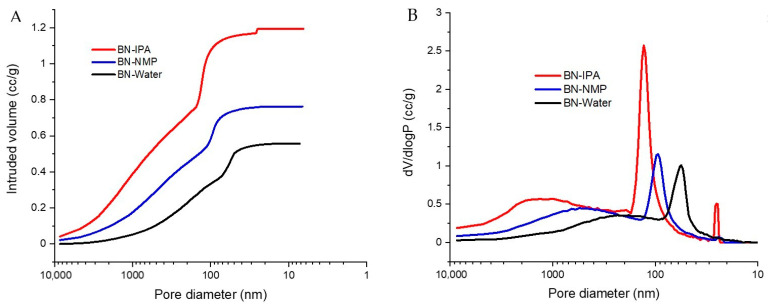
Comparison of mercury intrusion characteristics of the BN membrane samples (**A**,**B**) comparison of mercury pore size distributions of the BN membrane samples.

Mercury porosimetry was carried out to investigate porosity, in the approximate range of 10 nm to 10 µm. Figure 8A shows the intrusion of mercury into the membrane samples as a function of pressure, with pressure being analogous to pore diameter. As pressure is increased, the largest pores fill first, followed by increasingly smaller ones. For the BN-IPA sample, a very gradual filling is observed initially, for pores < 3 µm. A subsequent change in the slope of the curve corresponds to more rapid pore-filling. This continues until a sharp intrusion of mercury occurs (the slope rises sharply) as pores of approx. 130 nm diameter are filled. The curve then begins to plateau, before further minor uptake of mercury occurs, corresponding to 25 nm pores, after which the curve plateaus out as all pores are fully filled.

The curves observed for the BN-NMP and BN-Water samples are of a similar shape to BN-IPA, but the intrusion profiles are shifted to the right, corresponding to smaller pore sizes. In terms of pore volume, the trend mimics that of the pore size, insofar as the BN-IPA sample has the largest pore volume (1.19 cm^3^/g) compared to BN-NMP (0.76 cm^3^/g) and BN-Water (0.56 cm^3^/g). It is interesting to note that the pore volume of the BN-IPA sample is approximately double that of BN-Water—this implies that although the overall membrane morphologies have similarities (as evidenced by the similarity in shape of the intrusion curves), BN-Water, and to a lesser extent, BN-NMP, comprise more compact morphologies and have higher bulk densities relative to BN-IPA.

It is also useful to consider the pore size distributions of the membranes, plotted as pore diameter vs. the change in intruded volume with respect to the log of change in pressure (dV/dlogP), as shown in Figure 8B. When plotted this way, the pore size distributions can be evaluated in terms of sharpness, with sharp peaks corresponding to tight pore size distributions, and vice versa.

For BN-IPA, a broad peak is initially observed, indicative of pores in the approximate range of 0.4–4 µm. Broad peaks are also observed for the BN-NMP and BN-Water samples; these peaks are shifted to the right, relative to BN-IPA, corresponding to smaller pore sizes. A clear trend exists, therefore, in terms of micrometre- and sub-micrometre-sized pores being present and shifting to smaller sizes in the order IPA → NMP → water.

A second and more significant characteristic of Figure 8B is the presence of sharp peaks in the sub-200 nm range. For BN-IPA, a major peak occurs at 130 nm. The sharpness of this peak is indicative of a high concentration of pores, all of which are of a similar size. Less prominent peaks are observed for BN-NMP and BN-Water, and it is noted that these peaks shift to smaller pore sizes. The presence of a minor, secondary peak for the BN-IPA sample, corresponding to pores of 25.3 nm diameter, is also worth noting.

It is clear from the shape of the pore size distribution curves that the membranes studied here are derived from the same parent material, but it is also clear that each membrane has unique porous characteristics, depending on which solvent is used to conduct the membrane preparation.

BET analysis was carried out to further investigate the porous characteristics, specifically the surface area, pore diameter and pore volume. The measured data are summarised in Table S3.

Taking the surface area data, we observe values of 20.1, 12.9 and 26.7 m^2^/g for BN-IPA, BN-NMP and BN-Water, respectively. The largest surface area for BN-Water can be attributed to the fact that this sample has a high concentration of “small pores” (less than 50 nm in diameter) relative to the other two samples, as observed in Figure 8B above. BN-IPA has the largest pore size (130 nm) and thus might reasonably be expected to have the lowest surface area, but in fact, it has a value between that of BN-Water and BN-IPA. This can be attributed to the secondary porosity peak observed at 25.3 nm, on the basis that this subset of mesopores generates sufficient surface area to elevate the surface area value of BN-IPA above that of BN-NMP. In the literature, there is an established relationship between surface area and pore size; as pore size decreases, surface area increases, and generally speaking, as pore volume increases, surface area increases [72]. It must be remembered that the BET surface area value applies to the sample as a whole, and includes all surfaces and macroporosity, whereas the BJH pore diameter and pore volume values presented here are restricted to pores < 30 nm only.

In terms of pore diameter, there is a clear distinction between the BN-IPA (28.8 nm), BN-NMP (3.6 nm) and BN-Water (3.5 nm) samples; the diameter of BN-IPA is an order of magnitude greater. This is significant in the context of the porosimetry data, where the BN-IPA sample was the only sample to yield a peak sub-30 nm, i.e., a peak falling within the BET measurement range. Clearly, the BN-IPA sample has a subset of mesopores, which are absent for the BN-NMP and BN-Water samples. It is further noted that the BJH mean pore diameter value of 28.8 nm determined for the BN-IPA sample is in agreement with the value of 25.3 nm measured using mercury porosimetry.

The BN-NMP sample has the lowest pore volume. At 0.021 cm^3^/g, the pore volume is just under one-third that of BN-IPA and less than a quarter that of BN-Water. This very low pore volume may be a contributing factor to BN-NMP’s low surface area value relative to BN-IPA and BN-Water. Clearly, however, the porous characteristics of these membrane samples are overwhelmingly dominated by macroporosity, as evidenced by both mercury porosimetry data and microscopic imaging.

Excellent agreement has been established between the membranes’ physical porous properties and the corresponding morphologies, as imaged using electron microscopy. The SEM images (Figure 7) demonstrate a clear hierarchy in terms of the packing factor, in the order IPA → NMP → water, where BN-IPA could be described as “loosely packed” and BN-Water as “tightly packed”. This morphological difference is most evident from the images shown in Figure 7E,H, where BN-IPA displays obvious porosity (black regions in the image) and BN-Water could be perceived as non-porous (it appears grey, with low contrast across the image). Porosity can clearly be observed in the BN-IPA sample, with openings between the flakes in the order of 0.5–2 µm. A noticeable difference in feature size is also apparent, particularly between the BN-IPA and BN-Water samples, with BN-Water exhibiting a much finer texture and more compact arrangement of the BN flakes.

The “loosely packed” BN-IPA morphology is depicted by mercury porosimetry in the form of a broad peak in the approximate range of 0.4–4 µm as well as a high pore volume (1.19 cm^3^/g). The physical porous characteristics of BN-NMP and BN-Water are also consistent with the imaged morphologies observed in Figure 7. The sample with the least obvious porosity when viewed under SEM is BN-Water. However, the true porous nature of this sample was successfully captured by a combination of mercury porosimetry and BET analysis. The high surface area (26.7 m^2^/g) is attributed to the network of 50 nm pores, coupled with a reasonably high pore volume (0.56 cm^3^/g). These properties are characteristic of a low bulk density, nanoporous material having a predominantly open, interconnected porous network.

### 3.3. BN Membrane Testing

**Figure 9:**
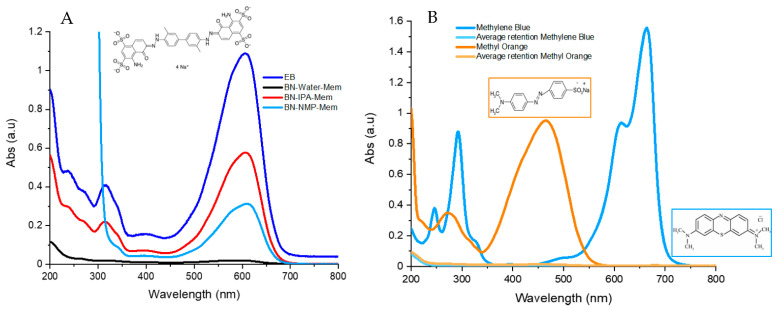
UV-Vis spectra showing the average retention of (**A**) 20 mL of Evans blue (15 µM) through BN membranes made from exfoliation in NMP, IPA and Millipore water. (**B**) Methyl orange (50 µm) and methylene blue (27 µm) through BN membranes exfoliated in Millipore water.

Three types of BN membranes, BN-NMP, BN-IPA and BN-Water, were produced. The thickness of the membranes and comparative dye retention data are shown in Table S4. The membranes were first tested using a standard dye, Evans blue [62], at a concentration of 15 µM and volume of 20 mL, with average results given in Figure 9A; the full values of retention are presented in the Supplementary Table S5 and further spectra are shown in Figure S16. An image of a membrane before and after passing Evans Blue through is shown in Figure S15. The BN-NMP and BN-IPA membranes showed similar retention values, with values of 72 ± 4% for NMP and 54.663 ± 12.015% for IPA (Figure 9A). In contrast, BN-Water showed far higher retention, with a mean of 98.4 ± 0.8% (Figure 9A). This result was highly reproducible, with very low standard deviation across the samples (Table S5), relative to the NMP and IPA membranes. The results obtained in water compare favourably with best reported results, such as those reported using MoS_2_ membranes (89%) [62] and results obtained for membranes produced from BN, which were functionalised by ball milling with urea [26]. However, our membranes have the advantage that no functionalisation is required and our retention values are comparable with similar dyes reported in the literature such as basic yellow [28,34,38], rhodamine B [73,74,75,76,77], Congo red [28,34,78] and malachite green [48], which showed retentions between 90% and 99%.

Therefore, due to the performance of the water-exfoliated BN-based membranes, a detailed investigation was carried out to determine the performance using two smaller dye molecules with different functionality, often utilised in retention studies: methyl orange and methylene blue. This also allows the universality of these results to be determined with nanofiltration needed to be effective for a wide range of possible molecules. The retention of these dyes (averaged spectra Figure 9B, further data can be found in Table S6 and Figure S17) showed values close to those obtained with Evans blue. Methyl orange showed a retention from 96% to 99 ± 0.882%, and the retention obtained with methylene blue was 99% to 99.9 ± 0.137%. As with the results of Evans blue, the samples showed appropriate repeatability (Table S6). Images of BN membranes after filtrating Methyl Orange and Methylene Blue are shown in Figure S18. The results obtained for methylene blue are higher or similar but over a longer period of time [48] than reported values in the literature for BN (95% retention), as well as functionalised BN (98% retention) [79]. Methyl orange is more often tested with other compounds [80,81], but it has been tested using functionalised BN with a negative charge, producing lower retention than the values reported here [39].

The retention and sorption mechanisms in BN structures are normally associated with π–π stacking interactions between the BN sheets and aromatic dye molecules, electrostatic interactions between the polar B-N bond and the charged and polar dyes, physisorption in the micropores and hydrophobic interactions. Here, we propose that BN retains the dyes via physisorption, and π–π stacking interactions take place between the BN rings and the aromatic rings present in the dye structures [44,79]. This is supported by the results indicating that all three dyes show strong separation by the membranes regardless of the charge of the dye molecules. In addition, no dependence on separation is found relating to the size of the dyes. Overall, these results reinforce the strong case for applying water-exfoliated BN-based membranes in nanofiltration applications.

### 3.4. Discussion of Trends

The strong performance of the membranes produced from water-exfoliated BN can be explained by a number of factors. Firstly, as seen in Raman, TEM and STEM, water exfoliates BN very efficiently, producing a high amount of monolayered BN nanosheets. This is due to the partial hydroxylation of the BN caused by sonication in water. This sonication-assisted hydrolysis of the solvent has been credited with helping the separation and dispersion of the BN nanosheets, which would effectively improve the level of exfoliation of the sample [60]. Secondly, the packing of the water-exfoliated BN nanosheets in the produced membranes is distinct from the NMP- and IPA-exfoliated membranes. We believe the packing plays a key role in the performance of the membrane. This distinct packing of the water-exfoliated BN is also confirmed by XRD, where it can be seen as a difference in the intensity of the peak [69]. This packing must affect the formation of the pores or channels where the water and dye are passing through the membrane, resulting in a performance uplift. This is supported by data obtained from mercury porosimetry, in which the BN-Water membranes have smaller pores than BN-NMP, whereas BN-IPA presents two subsets of pores, one being the biggest of the three cases. This small pore size could be a reason for BN-Water being more efficient in retaining the tested dye molecules. Moreover, data obtained from BET show a higher surface area and high pore volume for the BN-Water sample. This combination of properties provides better retention performance of the membrane, showing the characteristics of a nanoporous material with a predominantly open and interconnected porous network.

Therefore, overall, the performance of the BN-Water-Mem is linked to both the excellent degree of exfoliation obtained by exfoliating in water, the high surface area and small pore size, as well as the packing of the nanosheets; a combination that yields the best retention performance. Denser, more orderly packing of membranes has been reported to increase their performance [82,83], which is in line with what we found in our study.

## 4. Conclusions

Three selected solvents were effective in exfoliating BN and for producing corresponding BN-based membranes. It was found that solvent choice played a key role in membrane performance, with the use of water resulting in high levels of exfoliation and membranes with superior performance. The Raman peaks of water-exfoliated BN showed the largest shift compared to those of the bulk BN. TEM and STEM images showed the prevalence of monolayers to be highest in these samples. NMP also demonstrated very effective results for exfoliation, with AFM results showing clear steps in the deposited sheets, indicative of a highly exfoliated material in solution, with results showing no distinction between water and NMP in this case. In contrast, in all tests carried out, IPA showed the least degree of exfoliation.

Aspect-wise, the membranes from IPA and NMP showed similarities in terms of the packing of the nanosheets while the water-exfoliated membranes packed differently, in a much more efficient manner. The best retention-testing values were obtained for the water-exfoliated membrane, with pore size and surface area shown to play an important role, with smaller pores and high surface areas giving the best retention values. BN is thought to retain the dyes via physisorption and π–π interactions taking place between the BN rings and the aromatic rings present in the dye structures [44,84].

Overall, this work shows the potential that 2D-BN-based membranes offer for nanofiltration applications, such as water treatment and biopharmaceutical, food and agricultural separation technologies. Key to this is the choice of solvent used for exfoliation, with the cheapest and greenest of solvents, water, proving the best of those studied here.

## Supplementary Materials

The following supporting information can be downloaded at: https://www.mdpi.com/article/10.3390/nano12030473/s1, Table S1: Concentration and absorbance on the maximum of absorbance of the dyes; Figure S1: Size distribution of BN-IPA; Figure S2: Size distribution of BN in NMP; Figure S3: Size distribution of BN-Water; Figure S4: SEM images of Bulk BN; Figure S5: SEM images of nanosheets of BN-NMP; Figure S6: SEM images of nanosheets of BN-IPA; Figure S7: SEM images of nanosheets of BN-Water; Figure S8: STEM of 2D-BN produced in H_2_O (A–C), NMP (D–F) and IPA (G–H); Figure S9: TEM of 2D-BN produced in H_2_O (A–C), NMP (D–F) and IPA (G–H); Table S2: Main features of bulk and exfoliated BN in Raman; Figure S10: AFM images of dropcasted BN samples; Figure S11. FTIR spectra of exfoliated BN, BN-NMP (blue), BN-IPA (red), BN-Water (black) and BN-Bulk (green); Table S3: Summary of BET surface area analysis data; Figure S12: SEM (a) and (b) top view of the membranes and (c) and (d) cross-section of BN-NMP; Figure S13: SEM (a) and (b) top view of the membranes and (c) and (d) cross-section of BN-IPA; Figure S14: SEM (A) and (B) top view of the membranes and (C) and (D) cross-section of BN-Water; Figure S15: Picture of BN membrane (a) before and (b) after filtration with Evans Blue; Table S4. Retention and thickness of the membranes made from BN exfoliated, BN-NMP-Mem, BN-IPA-Mem and BN-Water-Mem; Figure S16: UV-Vis spectra of the retention of 20 mL of Evans Blue (15 µM) through BN mem-branes made from exfoliation in (a) NMP, (b) IPA,(c) Millipore water and (d) close up of the maximum absorbance peaks of the BN membranes from exfoliation in Millipore water; Table S5: Statistics from the retention of the membranes obtained with the different solvents, NMP, IPA and Millipore water; Figure S17: UV-Vis spectra of the retention of 20 mL of (a) Methyl Orange (50 µm) and (b) Methylene Blue (27 µm) through BN membranes exfoliated in Millipore water; Table S6: Statistics from the retention of the membranes obtained with Millipore water and tested with two dyes, Methyl Orange and Methylene Blue; Figure S18: BN membrane after filtration of (a) Methyl Orange and (b) Methylene Blue.
