# Supplementary material for: High-Performance Boron Nitride-Based Membranes for Water Purification
Source: Nanomaterials (Basel). 2022 Jan 29;12(3):473. doi: 10.3390/nano12030473 (PMC8838071; doi:10.3390/nano12030473)
Supplement: Supplementary file 1 [file nanomaterials-12-00473-s001.zip › nanomaterials-1543225-supplementary.pdf]

nanomaterials

MDPI

Supplementary Materials

High-Performance Boron Nitride Based Membranes for Water Purification

Natalia García Doménech [1,2], Finn Purcell-Milton [1,2], Adrián Sanz Arjona [1], Maria-Luisa Casasín García [1], Maeve Ward [1], Marc Brunet Cabré [1], Aran Rafferty [1], Kim McKelvey [3], Peter Dunne [1] and Yurii K. Gun'ko [1,2,*]

[1] School of Chemistry, Trinity College Dublin, D02 PN40 Dublin, Ireland; garciadn@tcd.ie (N.G.D.); PURCELFI@tcd.ie (F.P.-M.); SANZARJA@tcd.ie (A.S.A.); maria.luisa.casasin@gmail.com (M.-L.C.G.); mward5@tcd.ie (M.W.); brunetcm@tcd.ie (M.B.C.); rafferta@tcd.ie (A.R.); p.w.dunne@tcd.ie (P.D.)

[2] BiOrbic, Bioeconomy Research Centre, University College Dublin, D04 V1W8 Dublin, Ireland

[3] School of Chemical and Physical Sciences, Victoria University of Wellington, Wellington 6012, New Zealand; kim.mckelvey@vuw.ac.nz

* Correspondence: igounko@tcd.ie

Table S1. Concentration and absorbance on the maximum of absorbance of the dyes.

Dye	Concentration (μM)	Max. Abs (nm)	Absorbance (a.u)
Evans Blue	15	600	1
Methylene Blue	27	663	1.5
Methyl Orange	50	445	1.2

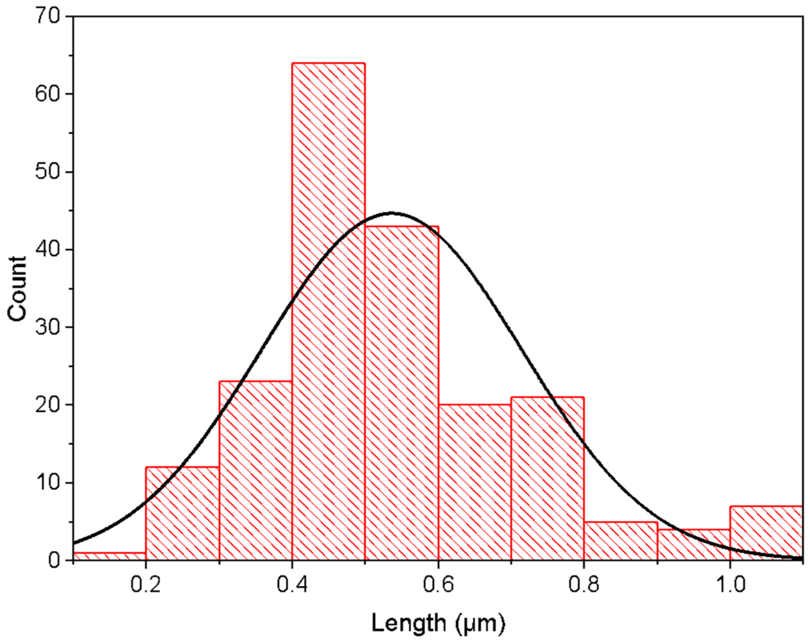

Figure S1. Size distribution of BN-IPA (in μm). Average length 0.536 ± 0.180 μm, N = 200.

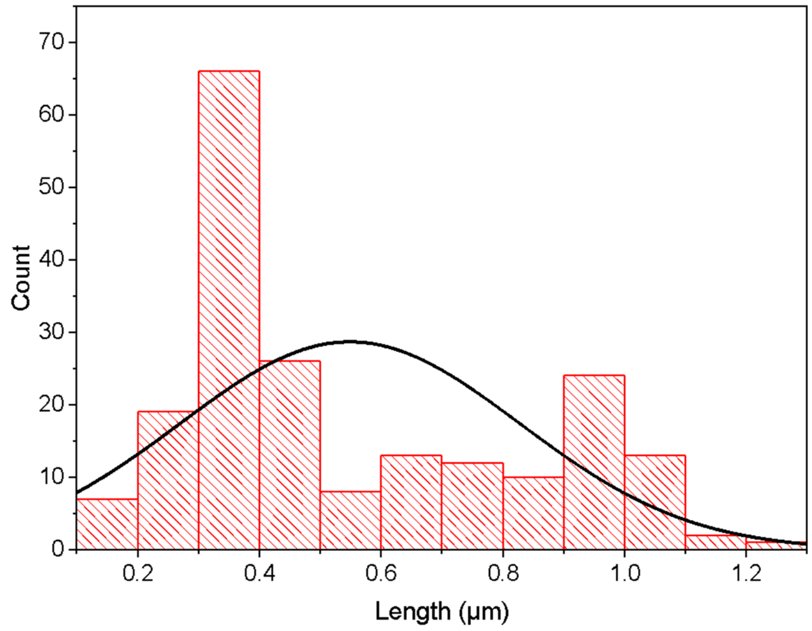

Figure S2. Size distribution of BN in NMP (in µm).). Average length 0.548 ± 0.280 µm, N = 200.

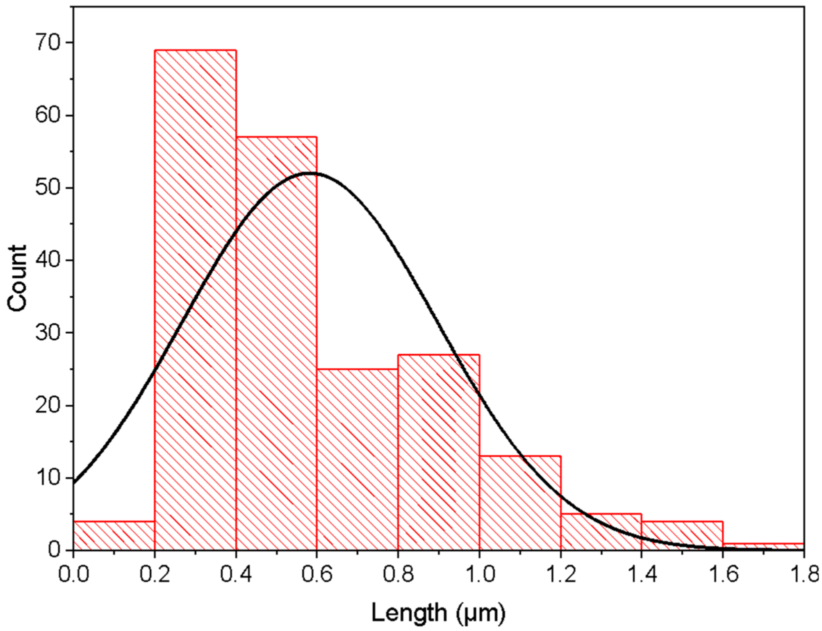

Figure S3. Size distribution of BN-Water (in µm). Average length 0.581 ± 0.314 µm, N = 200.

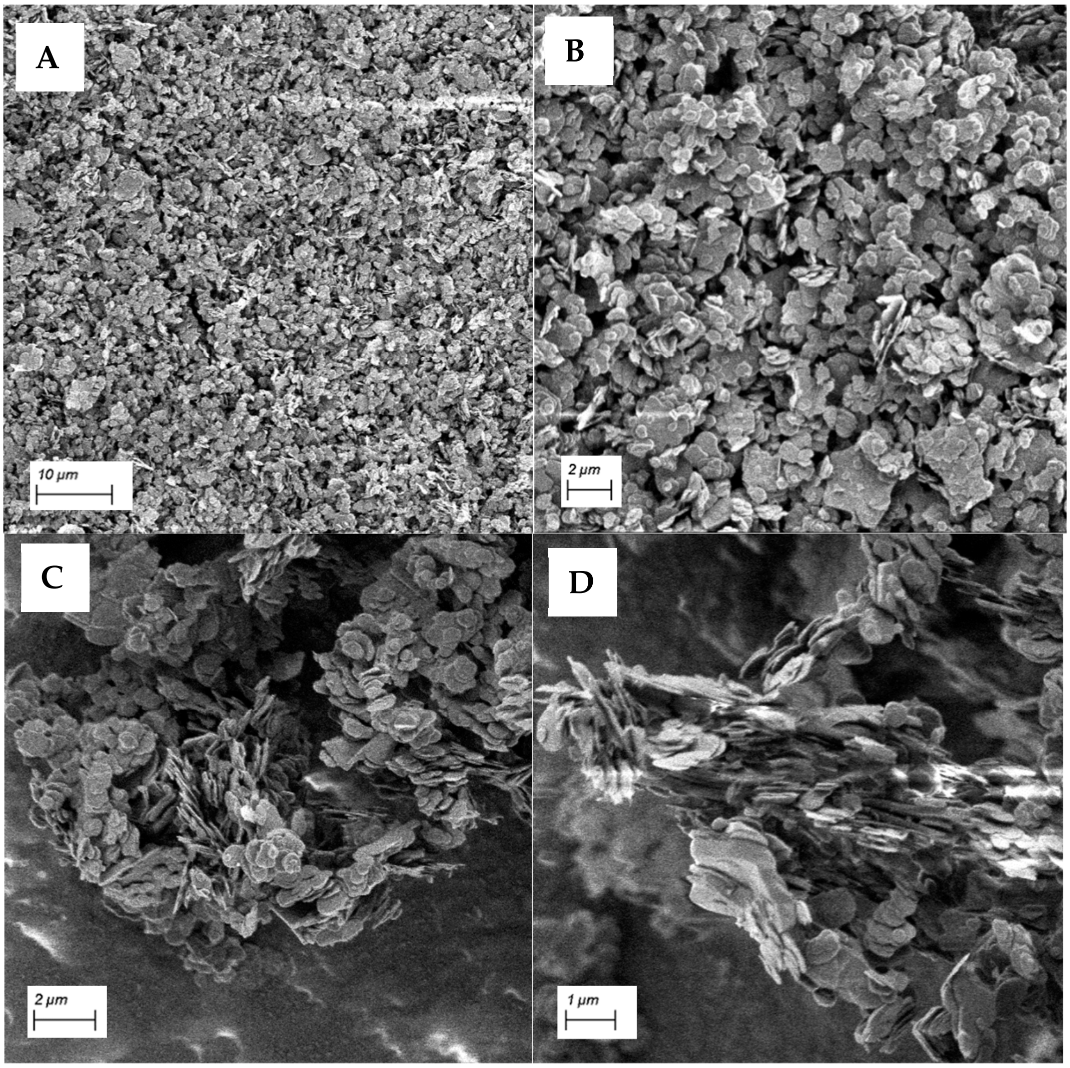

Figure S4. SEM images of Bulk BN.

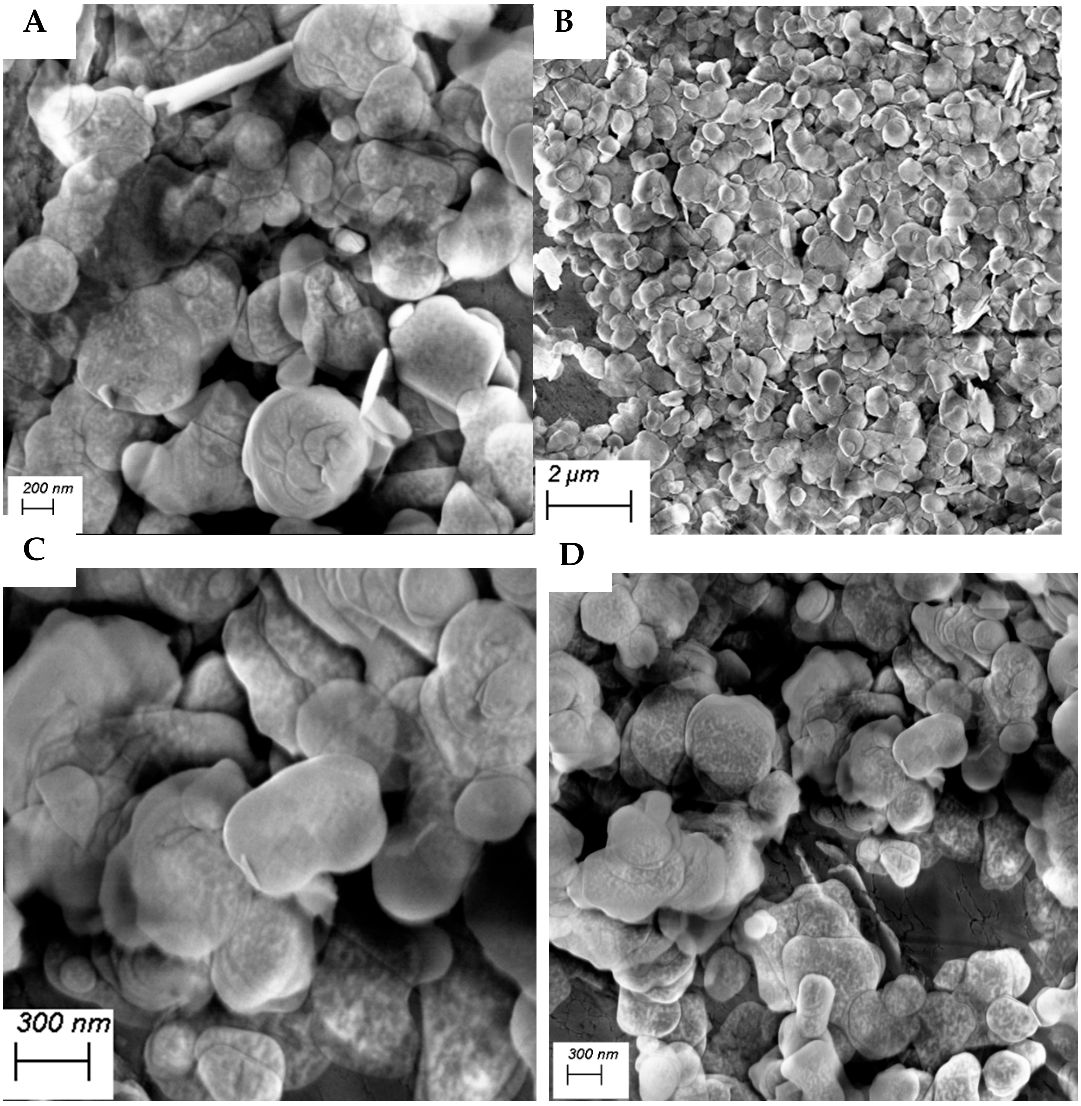

Figure S5. SEM images of nanosheets of BN-NMP.

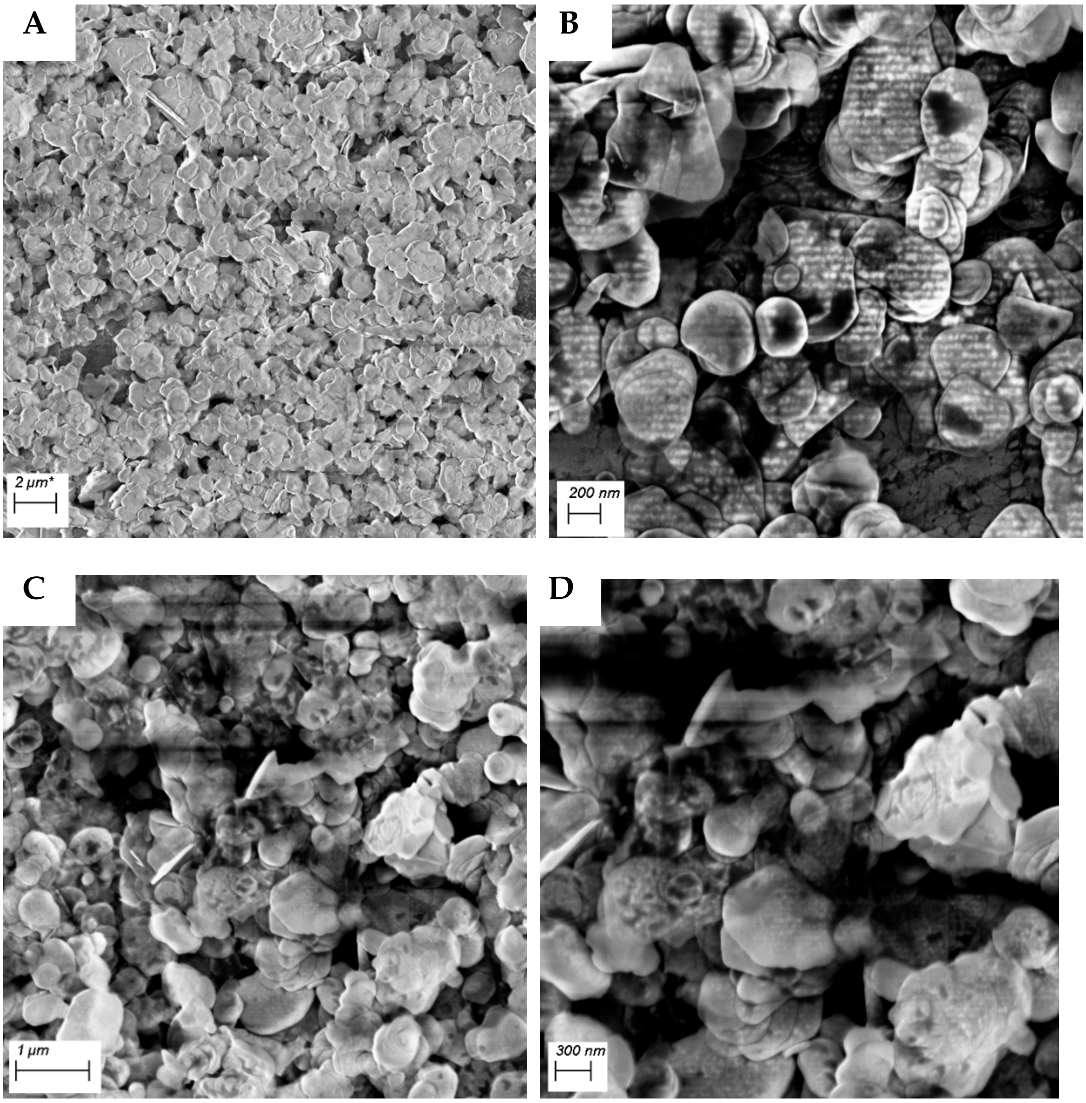

Figure S6. SEM images of nanosheets of BN-IPA.

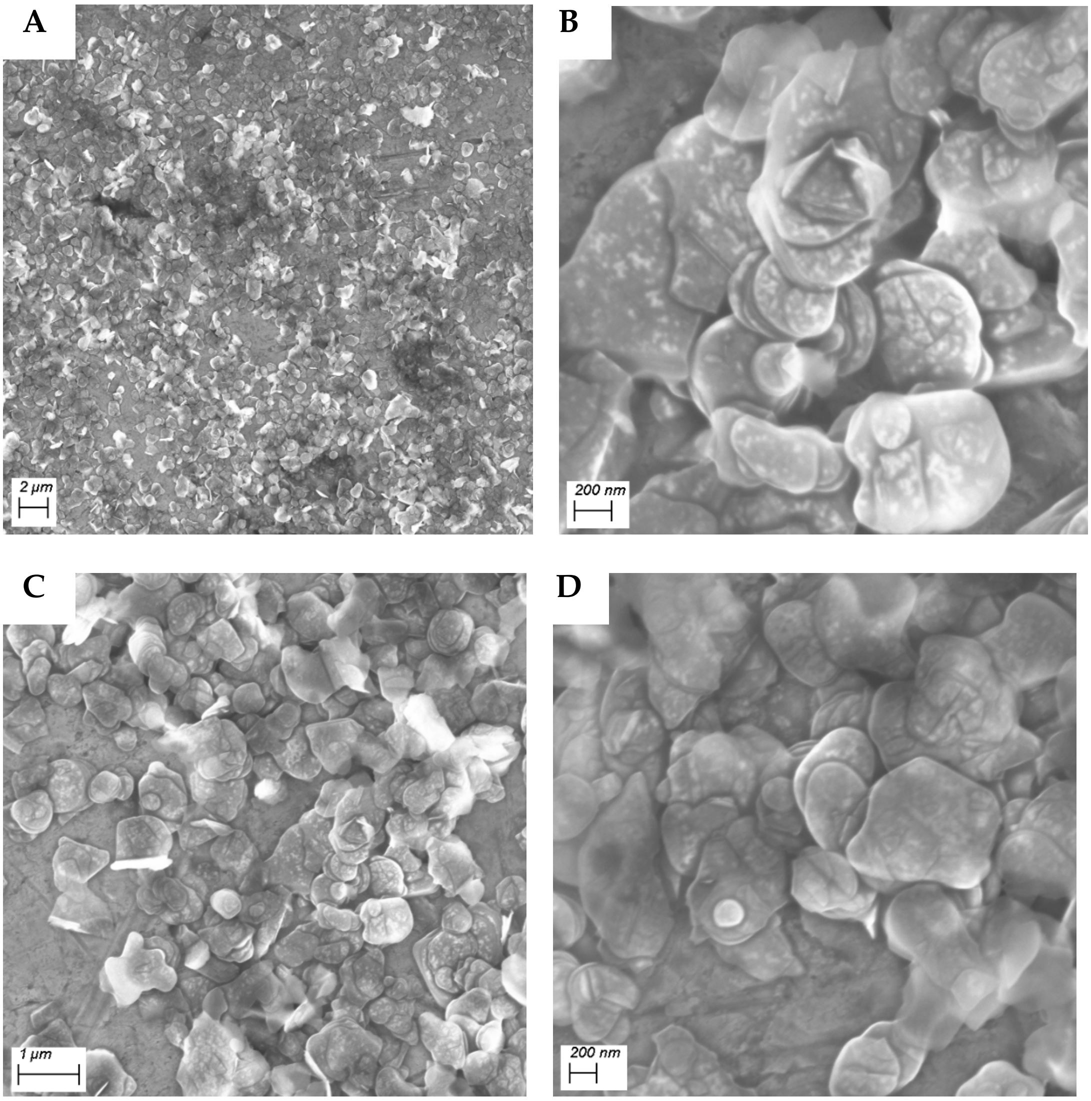

Figure S7. SEM images of nanosheets of BN-Water.

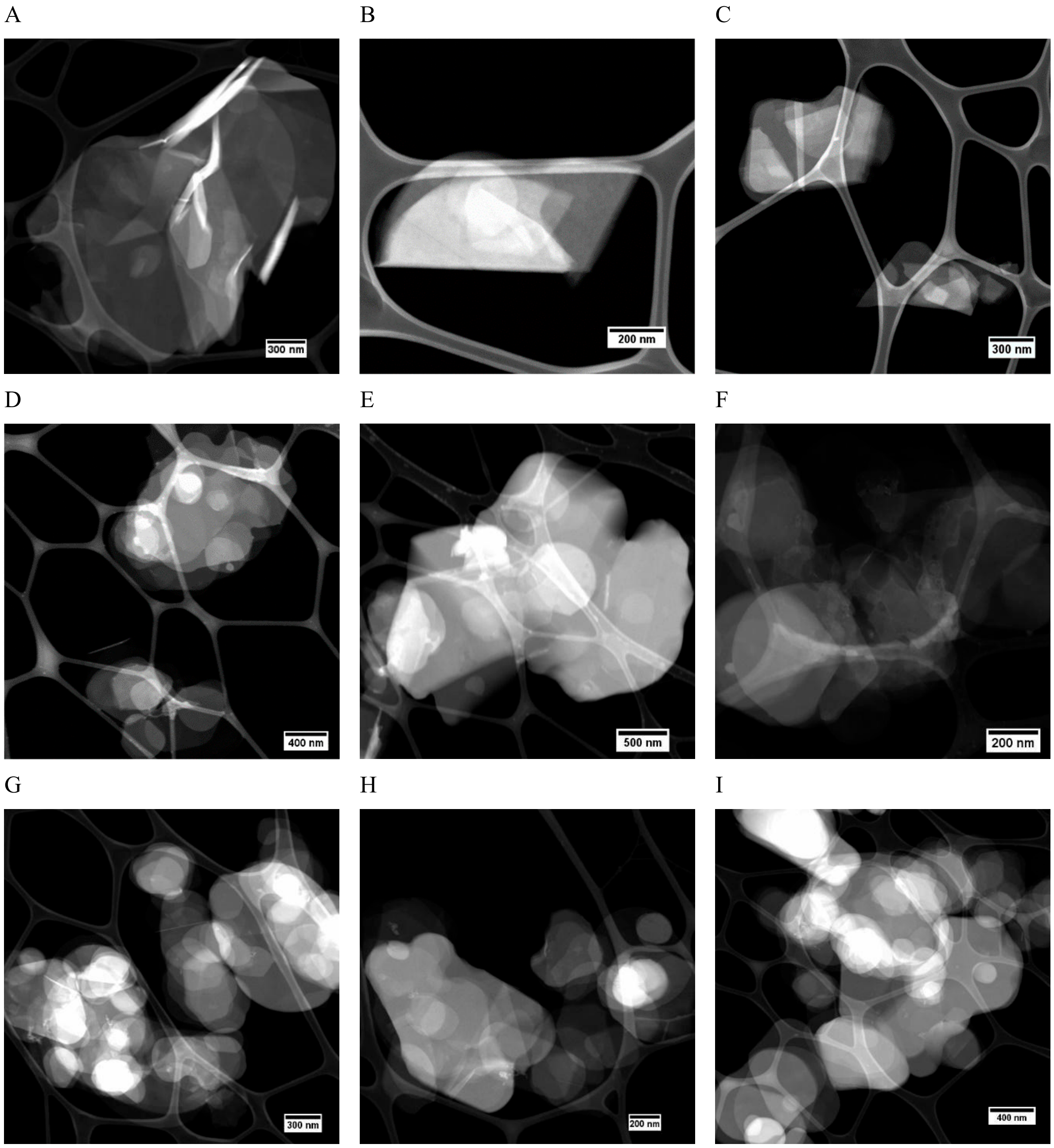

Figure S8. STEM of 2D-BN produced in H_2O (**A**–**C**), NMP (**D**–**F**) and IPA (**G**–**H**).

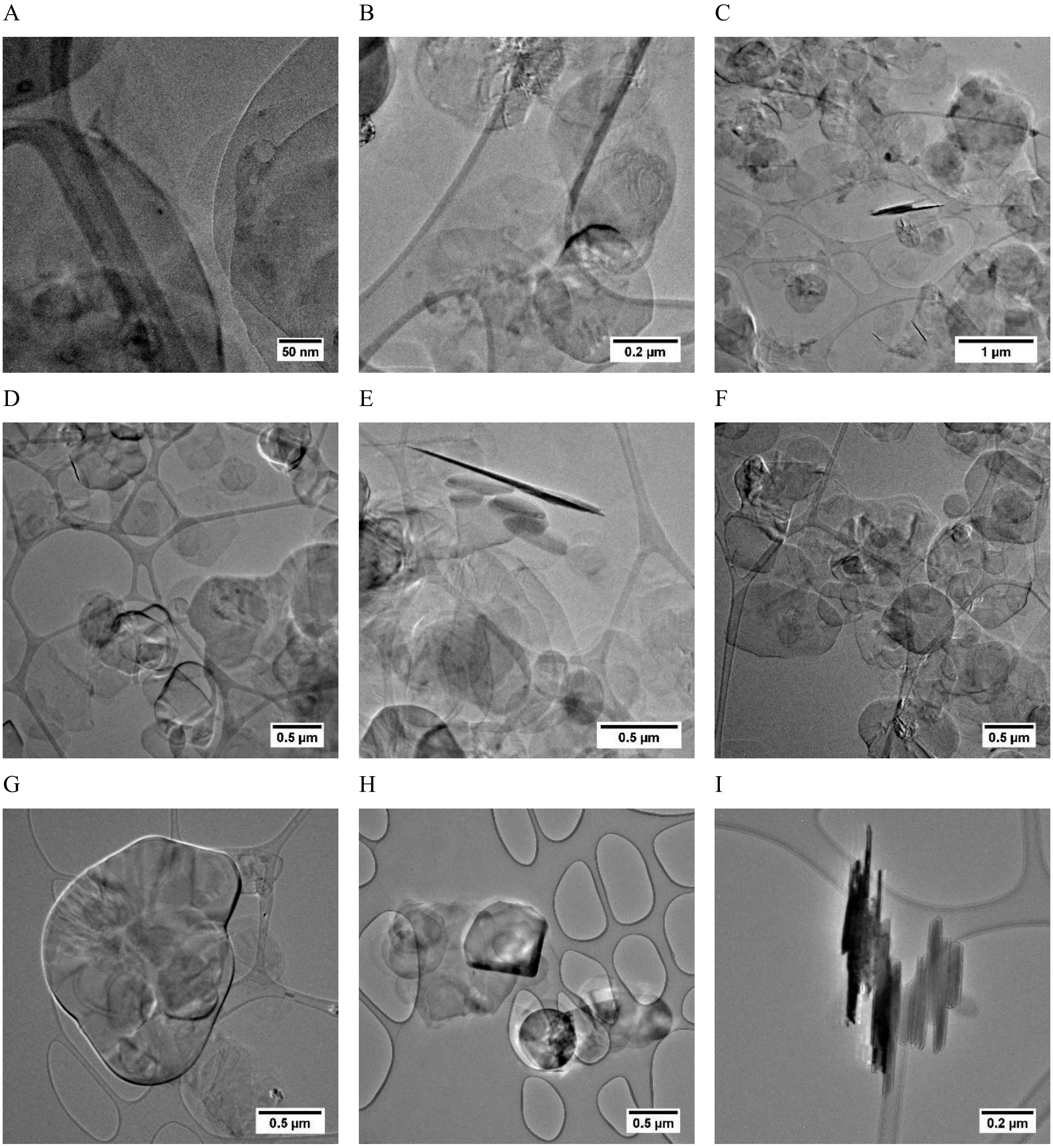

Figure S9. TEM of 2D-BN produced in H_2O (**A**–**C**), NMP (**D**–**F**) and IPA (**G**–**H**).

Table S2. Main features of bulk and exfoliated BN in Raman.

Sample	**Peak Shift (cm^{-1})**	**Intensity (a.u.)**	**FWHM (cm^{-1})**
**BN-Bulk**	1366.96	32077.4	9.37
**BN-NMP**	1367.85	12502.7	9.48
**BN-IPA**	1367.85	8884.2	9.36
**BN-Water**	1367.85	7248.1	10.05

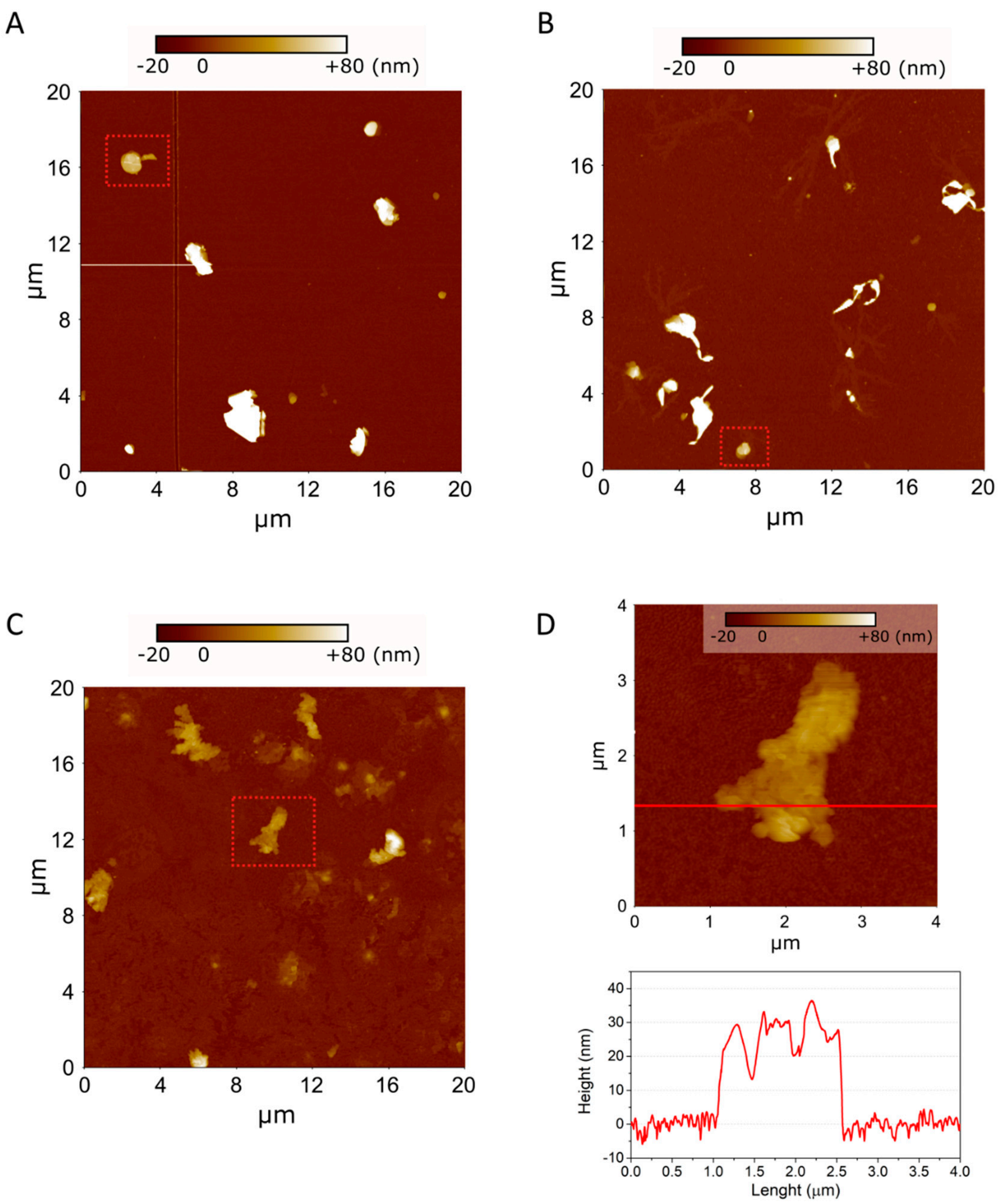

Figure S10. AFM images of dropcasted BN samples. **(A)** BN-H_2O **(B)** BN-NMP and **(C)** BN-IPA, with red square indicating magnified area for higher resolution AFM, as displayed on Figure 8 A, Figure 8 B, and Figure S15 D. (**D)** AFM image of single flake of BN prepared using IPA and height profile of line marked in red.

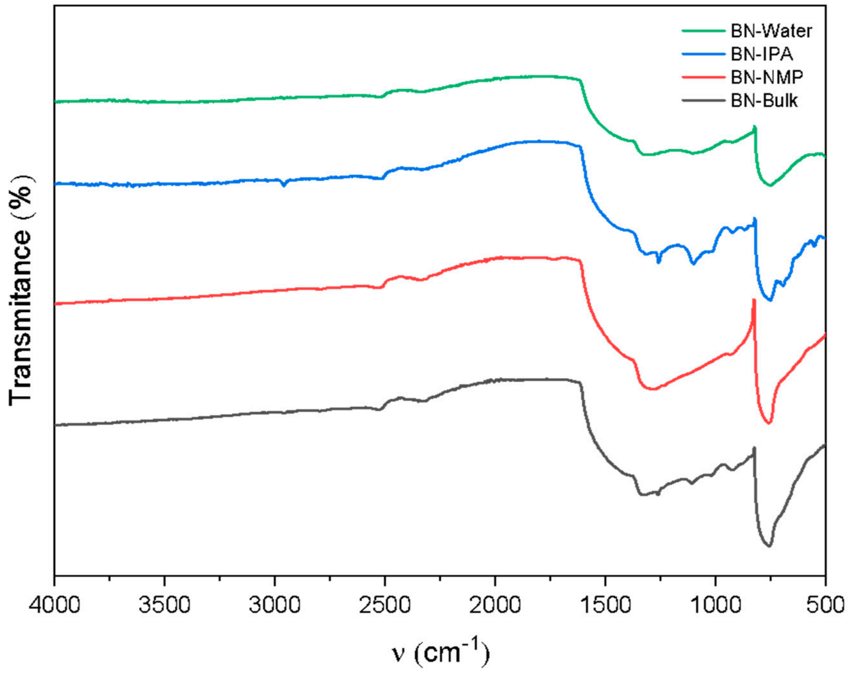

Figure S11. FTIR spectra of exfoliated BN, BN-NMP (blue), BN- IPA (red), BN-Water (black) and BN-Bulk (green).

Table S3. Summary of BET surface area analysis data.

Sample	Surface area (m^2/g)	BJH desorption pore diameter (nm)	BJH desorption pore volume (cm^3/g)
BN-IPA	20.1	28.8	0.055
BN-NMP	12.9	3.6	0.021
BN-Water	26.7	3.5	0.089

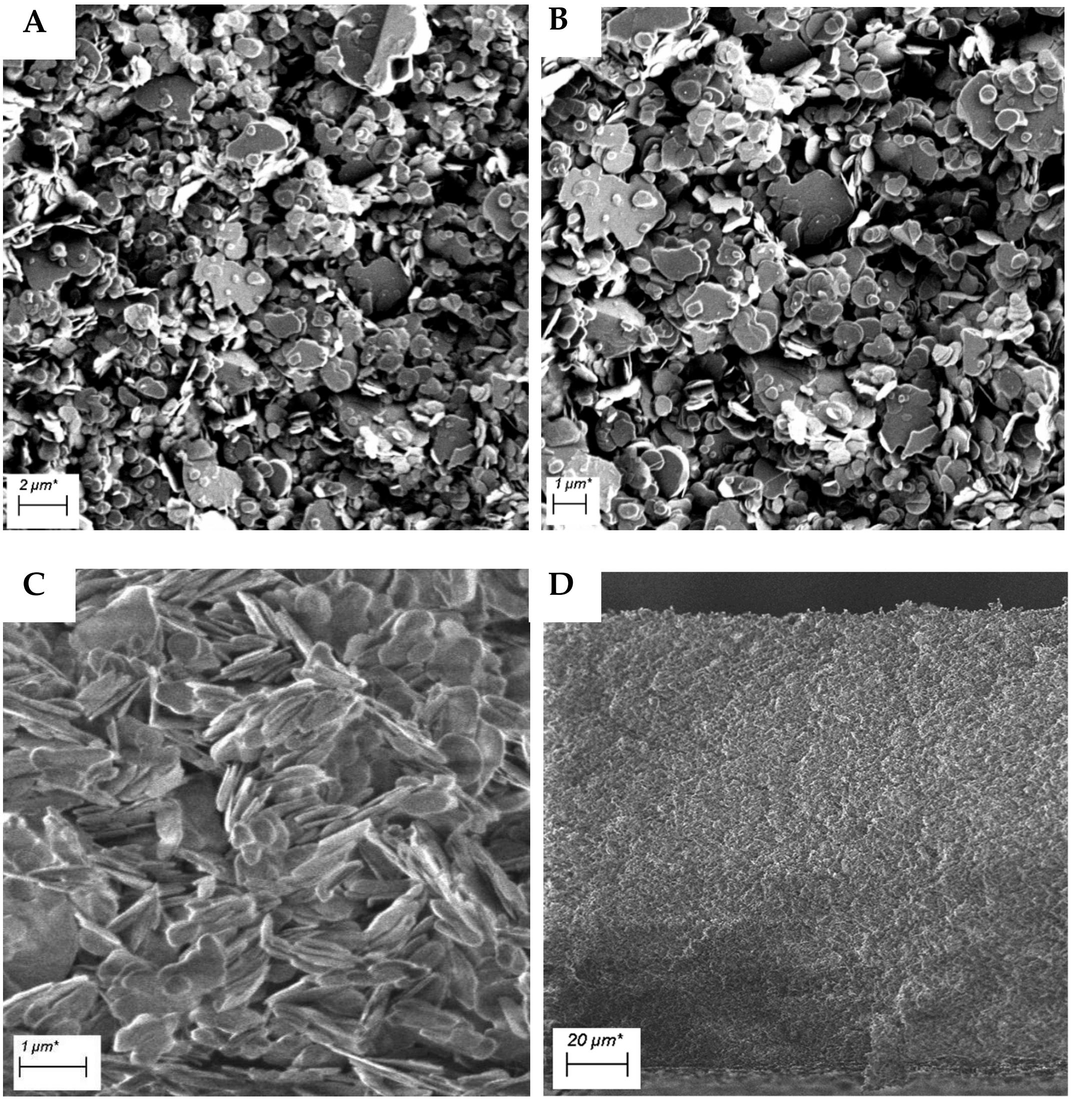

Figure S12. SEM (**a**) and (**b**) top view of the membranes and (**c**) and (**d**) cross-section of BN-NMP.

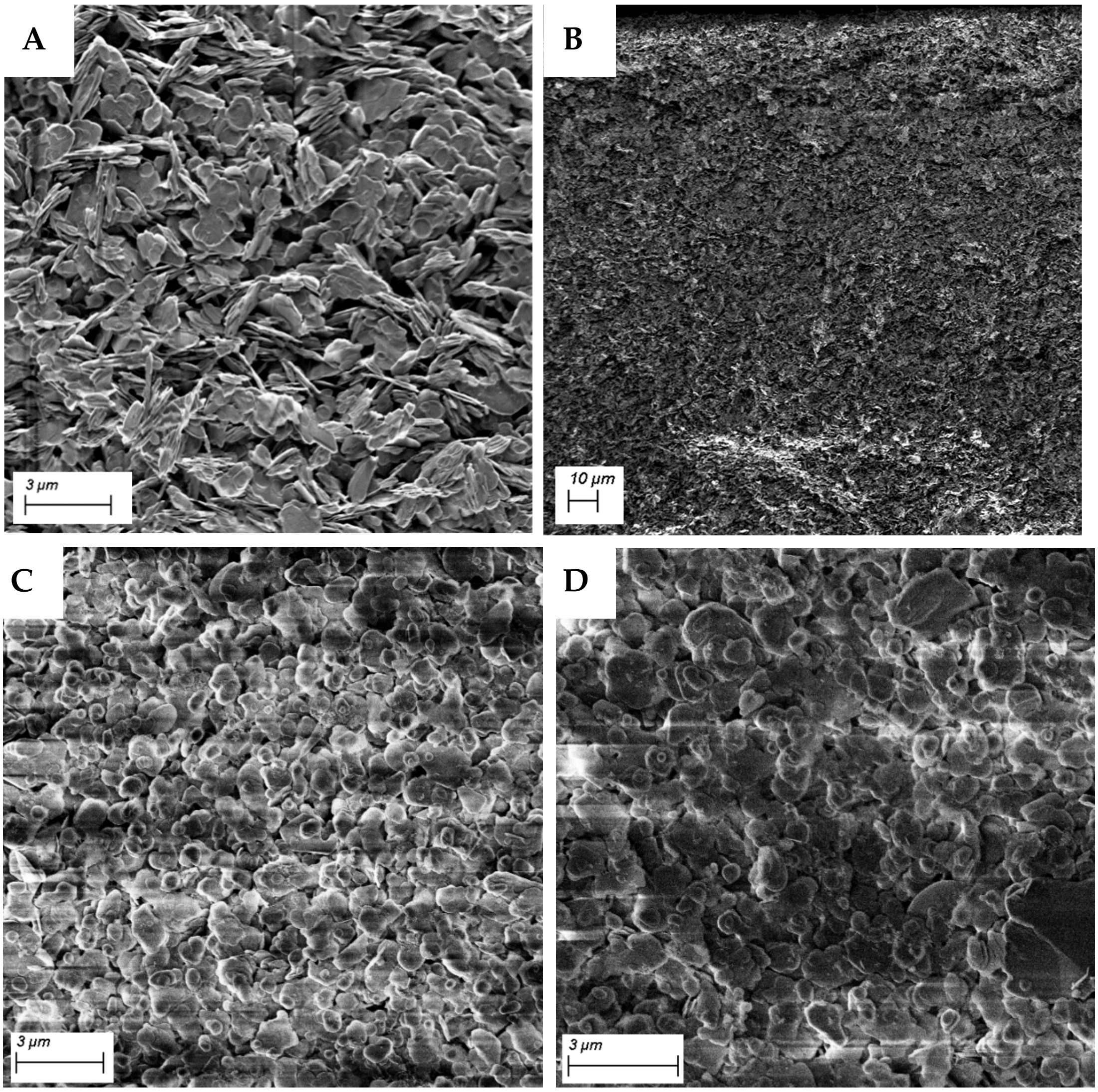

Figure S13. SEM (**a**) and (**b**) top view of the membranes and (**c**) and (**d**) cross-section of BN-IPA.

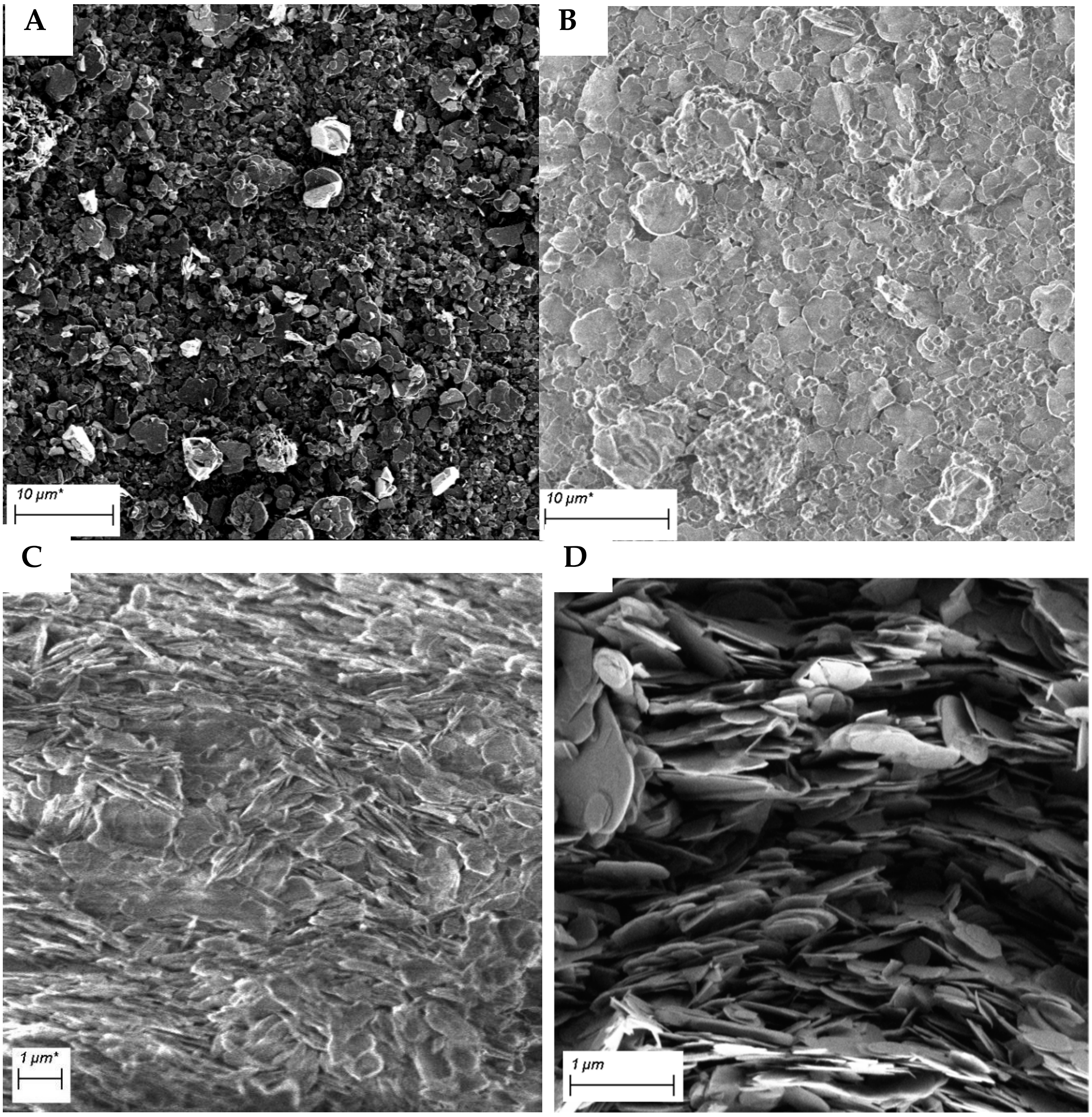

Figure S14. SEM (**A**) and (**B**) top view of the membranes and (**C**) and (**D**) cross-section of BN-Water.

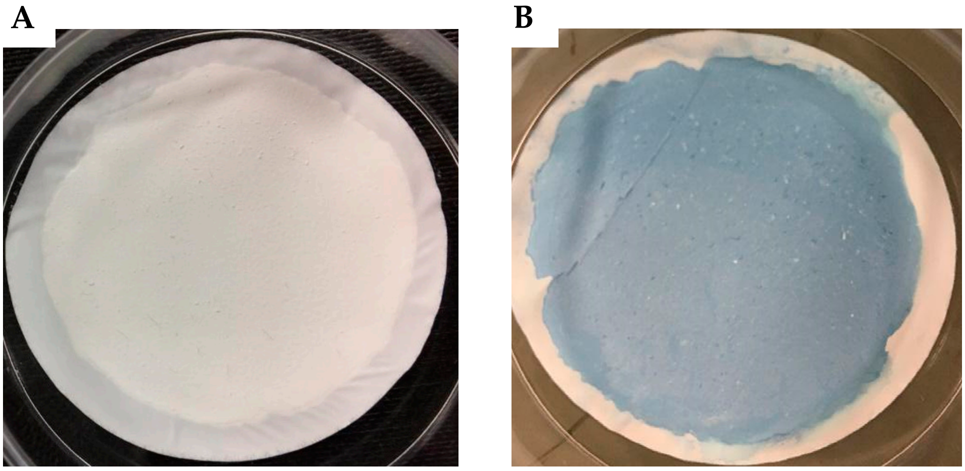

Figure S15. Picture of BN membrane (**a**) before and (**b**) after filtration with Evans Blue.

Table S4. Retention and thickness of the membranes made from BN exfoliated, BN-NMP-Mem, BN-IPA-Mem and BN-Water-Mem.

Sample	Retention (%)	Thickness (μm)
BN-NMP	72.88	148.90
BN-IPA	59.66	174.40
BN-Water	98.43	142.20

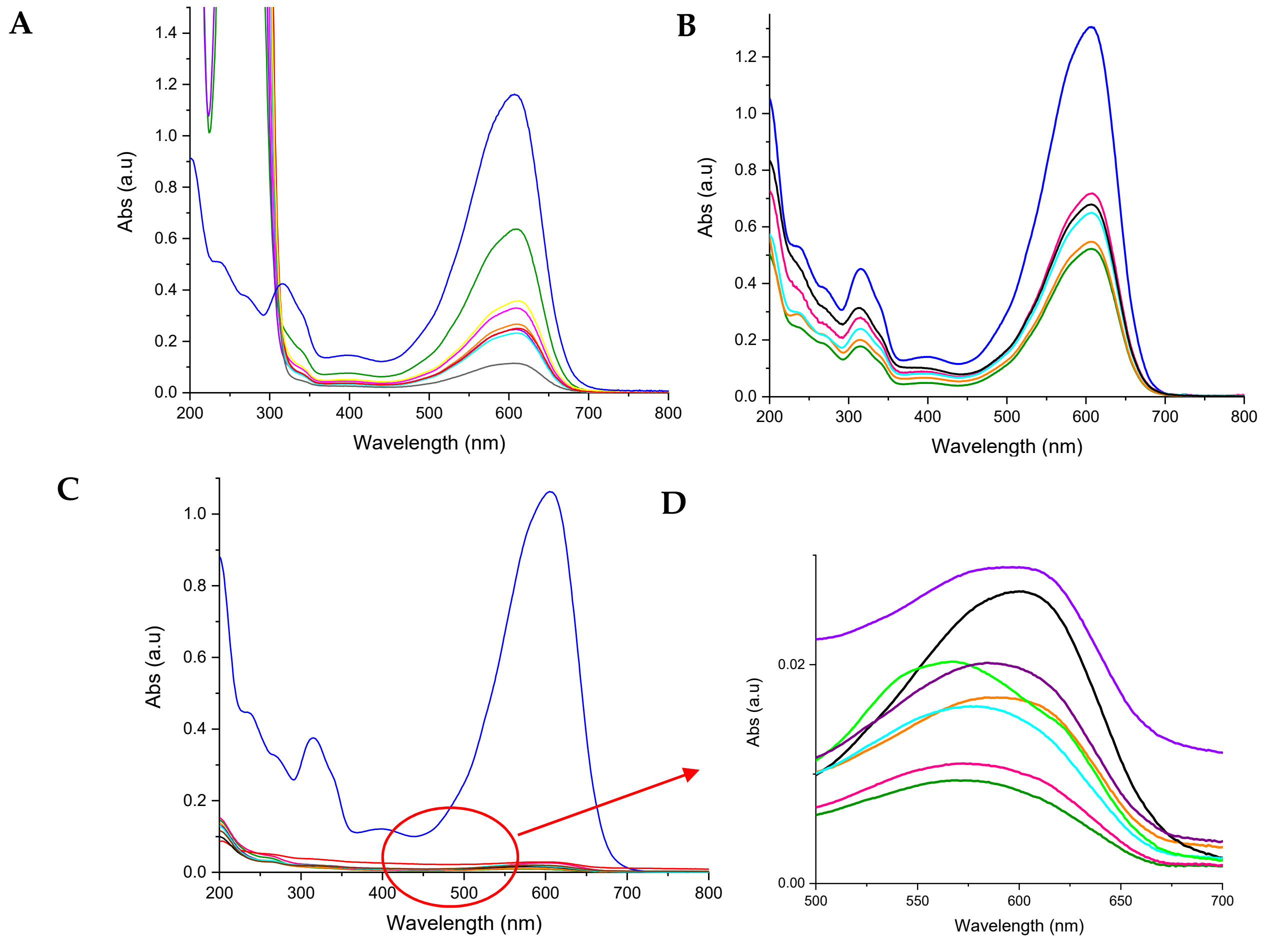

Figure S16. UV-Vis spectra of the retention of 20 mL of Evans Blue (15 µM) through BN membranes made from exfoliation in (**a**) NMP, (**b**) IPA,(**c**) Millipore water and (**d**) close up of the maximum absorbance peaks of the BN membranes from exfoliation in Millipore water.

Table S5. Statistics from the retention of the membranes obtained with the different solvents, NMP, IPA and Millipore water. The values where calculated using Origin 2018.

Solvent used	N total	Mean	Standard Deviation	Minimum	Median	Maximum
NMP	14	72.884	4.064	67.300	71.600	80.020
IPA	13	54.663	12.015	41.220	50.826	83.571
Millipore water	30	98.428	0.763	96.733	98.642	99.417

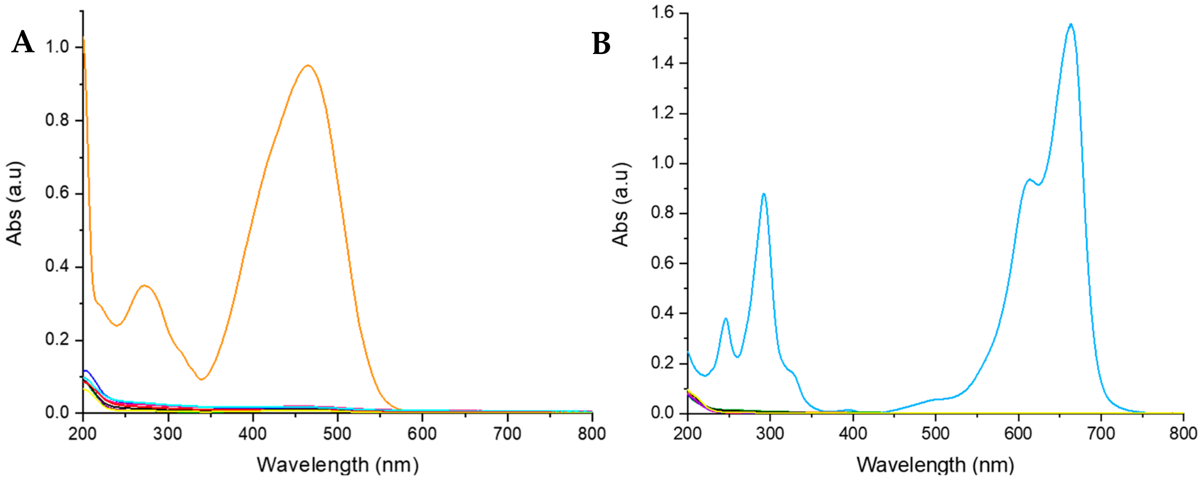

Figure S17. UV-Vis spectra of the retention of 20 mL of (**a**) Methyl Orange (50 µm) and (**b**) Methylene Blue (27 µm) through BN membranes exfoliated in Millipore water.

Table S6. Statistics from the retention of the membranes obtained with Millipore water and tested with two dyes, Methyl Orange and Methylene Blue. The values were calculated using Origin 2018.

Dye tested	N total	Mean	Standard Deviation	Minimum	Median	Maximum
**Methyl Orange**	16	98.850	0.882	96.839	98.960	99.872
**Methylene Blue**	23	99.856	0.137	99.447	99.892	99.978

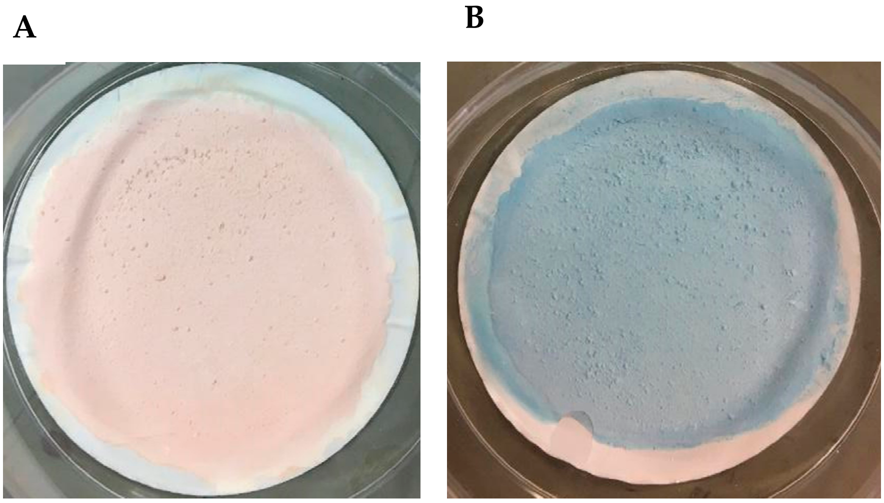

Figure S18. BN membrane after filtration of (**a**) Methyl Orange and (**b**) Methylene Blue.
